# Escalating impacts of climate extremes on critical infrastructures in Europe

**DOI:** 10.1016/j.gloenvcha.2017.11.007

**Published:** 2018-01

**Authors:** Giovanni Forzieri, Alessandra Bianchi, Filipe Batista e Silva, Mario A. Marin Herrera, Antoine Leblois, Carlo Lavalle, Jeroen C.J.H. Aerts, Luc Feyen

**Affiliations:** a European Commission, Joint Research Centre, Directorate for Sustainable Resources, I-21027, Ispra, Italy; b Arcadia SIT, I-27029, Vigevano, Italy; c European Commission, Joint Research Centre, Directorate for Growth and Innovation, I-21027, Ispra, Italy; d Arhs Developments, L-1253, Luxembourg; e Laboratoire d'Économie Forestière (LEF), AgroParisTech, INRA, 54000, Nancy, France; f Amsterdam Global Change Institute (AGCI), Vrije Universiteit Amsterdam, 1081 HV Amsterdam, The Netherlands; g Institute for Environmental Studies (IVM), Vrije Universiteit Amsterdam, 1081 HV Amsterdam, The Netherlands; h European Commission, Joint Research Centre, Directorate for Space, Security and Migration, I-21027, Ispra, Italy

**Keywords:** Multiple climate hazards, Climate change impact, Loss and damage, Critical infrastructures

## Abstract

Extreme climatic events are likely to become more frequent owing to global warming. This may put additional stress on critical infrastructures with typically long life spans. However, little is known about the risks of multiple climate extremes on critical infrastructures at regional to continental scales. Here we show how single- and multi-hazard damage to energy, transport, industrial, and social critical infrastructures in Europe are likely to develop until the year 2100 under the influence of climate change. We combine a set of high-resolution climate hazard projections, a detailed representation of physical assets in various sectors and their sensitivity to the hazards, and more than 1100 records of losses from climate extremes in a prognostic modelling framework. We find that damages could triple by the 2020s, multiply six-fold by mid-century, and amount to more than 10 times present damage of €3.4 billion per year by the end of the century due only to climate change. Damage from heatwaves, droughts in southern Europe, and coastal floods shows the most dramatic rise, but the risks of inland flooding, windstorms, and forest fires will also increase in Europe, with varying degrees of change across regions. Economic losses are highest for the industry, transport, and energy sectors. Future losses will not be incurred equally across Europe. Southern and south-eastern European countries will be most affected and, as a result, will probably require higher costs of adaptation. The findings of this study could aid in prioritizing regional investments to address the unequal burden of impacts and differences in adaptation capacities across Europe.

## Highlights

• Projections of multiple climate risks to critical infrastructures are assessed.
• Impacts could rise up to 10 times present damages by 2100 due to global warming alone.
• Damages from heatwaves, droughts and coastal floods show the most dramatic rise.
• Economic losses could be highest for the industry, transport and energy sectors.
• Southern and south-eastern European countries will likely be most affected.

## 1 Introduction

‘Critical infrastructures’ refers to the array of physical assets, functions, and systems that are vital to ensuring the European Union’s (EU’s) health, wealth, and security (European Council, 2008). According to this definition, they include existing transport systems, renewable and non-renewable energy generation plants, industry, water supply networks, and education and health infrastructures. The main threats presented by climate to infrastructure assets include damage or destruction from extreme events (Handmer et al., 2012), which climate change is expected to exacerbate (Fischer and Knutti, 2015, Pall et al., 2011, Rahmstorf and Coumou, 2011, Stott et al., 2004). Different types of infrastructures have different levels of vulnerability to climate change. Moreover, as climate change impacts are manifested locally, individual assets have different hazard exposures depending on their geographical location. Understanding and quantifying these risks is crucial for planning suitable adaptation measures to safeguard and secure the functioning of society.

Previous studies on sectorial impacts of climate change have focused mostly on single hazards or a limited set of hazards, so their estimates can only partially represent the potential consequences of future climate extremes (Arnell et al., 2013, Ciscar et al., 2011, Hsiang et al., 2017, Lung et al., 2013, Piontek et al., 2014, van Vliet et al., 2012). Furthermore, they usually refer to broad sectorial categories (e.g. water, agriculture), without providing information on the climate effects at infrastructure level, quantifying which is essential to develop climate-proofing measures for key societal services. Various impacts of climate extremes on infrastructures are acknowledged in the literature, but they are primarily presented in qualitative, descriptive terms (Cruz and Krausmann, 2013, Michaelides et al., 2014, Schaeffer et al., 2012). Quantifying the effects of climate hazards on infrastructures is a complex task because of incomplete scientific methodologies and limited understanding of vulnerabilities of infrastructures (Mechler et al., 2014, Neumann et al., 2014). Existing methods of assessing direct costs generally focus on specific hazards or sectors by the use of susceptibility curves derived analytically under specific conditions (Carleton and Hsiang, 2016, Ciscar et al., 2011, Meyer et al., 2013). However, such approaches showed large uncertainties due to the poor calibration on observed damage (Jongman et al., 2012). Difficulties in establishing comparisons across hazards and sectors remain particularly relevant (Kappes et al., 2012). Moreover, datasets of existing infrastructures are collected and maintained by various institutions (e.g. public or private) for different purposes and thus lack homogeneity in terms of spatial and thematic coverage and detail, semantics, format, and units of measurement. Harmonizing geo-data is essential to develop spatially coherent assessments of the potential impacts of natural hazards (Fekete et al., 2016); however, it remains challenging for continental-scale approaches given the relevant variety across and within datasets.

In this study we seek to fill the above-mentioned gaps by providing a comprehensive multi-hazard risk assessment of critical infrastructures in Europe under climate change and identifying the most affected regions throughout the 21st century. For this purpose, we developed a novel method that combines climate-related disaster records with a set of high-resolution projections of climate hazard, a detailed representation of sectorial physical assets, and their vulnerability to the hazards. We believe that our data-model integration approach adds significant value in the following ways:

1 We consistently assess how the seven most harmful climate-related extremes (heat- and cold waves, droughts, wildfires, river and coastal floods and windstorms) evolve in Europe in view of global warming. Previous assessments of the sectorial impacts of climate extremes focused mostly on single or a limited set of climate hazards.
2 We develop a detailed and spatially coherent representation of current sectorial physical assets and productive systems. This analysis enables us to investigate impacts at infrastructure level never reached in previous studies on sectorial impacts.
3 We derive a qualitative appraisal of the vulnerability of critical infrastructures to each hazard based on the combination of an extensive literature review and a survey run amongst ∼2000 experts. This represents the first attempt to fill a gap in the scientific knowledge and provides a tractable database for appraising and comparing sensitiveness of different types of infrastructures to climate hazards, a prerequisite for assessing multi-hazard/multi-sector climate change impacts.
4 We calibrate risk scenarios based on more than 1100 climate-related losses recorded in the most comprehensive public disaster database so that projections of expected annual damages (EADs) are strongly rooted on the observational records.
5 We provide an exploration of the potential costs of adaptation required to increase resilience against future climate hazards based on reported benefit-to-cost ratios reported in literature.

The integration of these elements provides a range of plausible estimates of future extreme climate-related risks for the current stock of European infrastructures.

The paper is structured as follows. Section 2 (Methods) presents the overall framework and describes each specific component, including climate hazards, exposure data collection and harmonization, climate sensitivity of critical infrastructures, risk integration and adaptation scenarios. Section 3 (Results) reports and discusses the overall multi-hazard multi-sector risks, the impacts at sector- and infrastructure level, including the spatial and temporal variability therein, and the costs of adaptation. This section further describes the main limitations of our study and knowledge gaps. Section 4 (Conclusions) synthesizes the key findings of this study and highlights challenges for future research.

## 2 Methods

### 2.1 Methodological framework

**Fig. 1:**
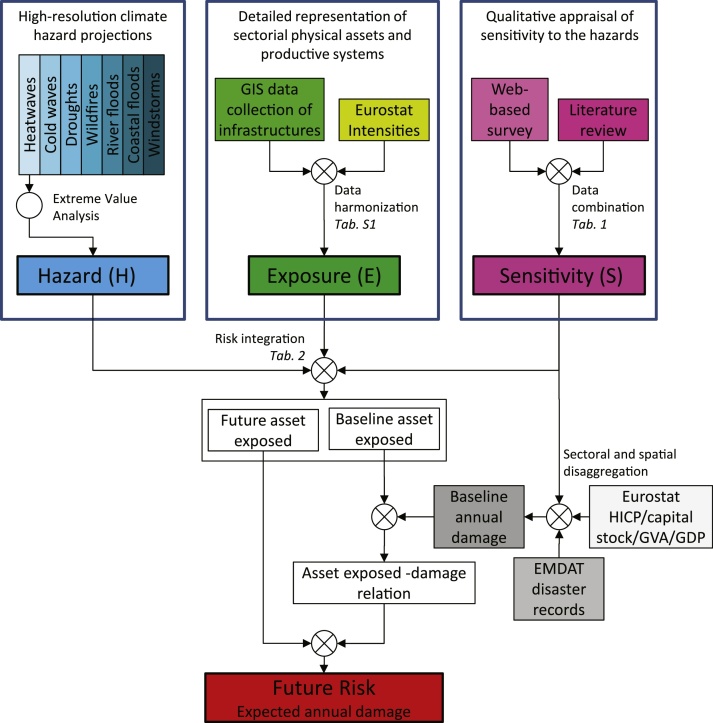
Schematic overview of the methodological approach. Components of hazard, exposure, and sensitivity are displayed in blue, green, and magenta, respectively, baseline annual damage (and related input datasets) in gray, and future risk of climate hazards in red. The flow diagram refers to a climate change scenario with static sensitivity and no changes in the distribution of infrastructures. Tables cited in italic (Table) are detailed in the main text and Supplementary material. (For interpretation of the references to colour in this figure legend, the reader is referred to the web version of this article.)

We employed the risk framework proposed by the IPCC (2014) to estimate the climate impacts as a combination of climate hazards (*H*), exposed infrastructures (*E*) and their sensitivity (*S*) to the hazards. The data-driven prognostic approach employed by Forzieri et al. (2017) to estimate human mortality due to multiple climate extremes has been further developed here to derive the susceptibility to climate hazards of critical infrastructures and to monetize consequent impacts. The methodology integrates a set of high-resolution climate hazard projections generated under a “business-as-usual” greenhouse gas emissions trajectory, a detailed representation of sectorial physical assets and productive systems, and a qualitative appraisal of their sensitivity to the hazards based on the combination of expert view and literature review. The three above-mentioned components are linked with more than 1100 records of climate disaster damage in order to derive a comprehensive and comparable set of climate hazard damage functions strongly based on observational records. Fig. 1 shows the methodological approach used in this work. Each of the risk components is visually represented in the figure by a different color and described in the following sections.

We present the multi-hazard impacts of future climate to the present stock of infrastructures in order to avoid hypotheses on changes in society up to the end of the century. Damage estimates cover the EU28 plus Switzerland, Norway, and Iceland (referred to herein as EU+) undiscounted and expressed in 2010 euros. Finally, based on literature-derived average benefit-to-cost ratios (BCRs), we provide an exploration of the possible costs of adaptation required to increase resilience against future climate hazards.

### 2.2 Climate hazards (*H*)

The analysis focuses on seven climate hazards, namely heat and cold waves, river and coastal floods, droughts, wildfires, and windstorms, derived for 1981–2010 (baseline), 2011–2040 (referred to as the 2020s for short), 2041–2070 (2050s) and 2071–2100 (2080s), for an ensemble of bias-corrected climate projections under the A1B emissions scenario (Table S1). The quantification of the hazard component is based on the analysis of the changes in frequency of extreme climate events proposed by Forzieri et al. (2016). Baseline return levels of the climate hazard indicators with return periods from 2 to 100 years were obtained at each 1-km grid cell by extreme value analysis, and corresponding future variations in frequency were calculated by inversion of the fitted probability functions. Hazard magnitude levels (*H_L_*) were classified based on the probability of occurrence of events in current climatology; given *T_R_* as return period corresponding to *H_L_* in today’s climate, we assigned the intensity class to the *H_L_* event as very high (*T_R_* ≥ 100yr), high (100yr > *T_R_* ≥ 50yr), moderate (50yr > *T_R_* ≥ 20yr), low (20yr > *T_R_* ≥ 10yr), very low (10yr > *T_R_* ≥ 2yr) or no hazard (2yr >*T_R_*). The fraction of a given area that is expected in the future to be annually exposed to a hazard of *H_L_* magnitude − hereafter labelled as *H* to simplify the notation − was derived for each intensity class by integrating the potential exposure to hazard events over the probability of occurrence. Thus, *H* inherently accounts for the future changes in frequency of the hazardous event. The significance of the changes in climate hazard was evaluated separately for each climate model by the Kolmogorov–Smirnov test applied on the annual values of future time windows versus baseline. For pixels with non-significant changes, we kept baseline *H* values for future time periods. This implies that the projections of impacts reported herein reflect only significant changes (*p*-value < 0.05) in hazards due to climate change. More details are available from Forzieri et al. (2016).

### 2.3 Exposure data collection and harmonization (*E*)

Exposure was described by a comprehensive set of geographic information system (GIS) vector layers that represent the current stock of energy, transport, industry, and social infrastructures (Table S2), including the following assets:

- energy sector: non-renewable energy production (coal/oil/gas/nuclear power plants), renewable energy production (biomass and geothermal/hydro/solar/wind power plants) and energy transport systems (electricity distribution/transmission and gas pipelines);
- transport sector: roads, railways, inland waterways, ports, and airports;
- industry sector: heavy industries (metal/mineral/chemical/refineries) and water/waste treatment systems;
- social sector: education and health infrastructures (e.g. schools and hospitals).

The data were preliminarily converted from vector to raster data structure with a 1-km cell size. In order to allow intra-sector comparability between types of infrastructures and overcome possible incompleteness, the gridded data were harmonized by assigning sector-specific intensity values obtained from Eurostat (average values over 2009–2013) and assumed to be correlated to the economic value of the asset and its productivity. The utilized intensity values are electricity produced/transported (kilotonnes of oil equivalent) for energy infrastructures; annual freight transported (kilotonnes) for transport infrastructures; annual turnover (million euro) for industry and total public expenditure (million euro) for social infrastructures. The harmonization procedure consisted of disaggregating the national intensity values of each infrastructure type to the cells where those infrastructures are located within the country, based on a set of local attributes (e.g. MW of installed capacity for energy production plants, number of potential users for social infrastructures; Table S2). For ports and airports, for which Eurostat data were available in detail, intensity values were univocally assigned to each local asset. The resulting harmonized data represent the infrastructure-level exposure layers (*E*). It is worth noting that the procedure minimized the impact of geospatial data incompleteness issues, as the total intensity of a given infrastructure type in a given country was preserved within that country. We assumed no changes in exposed infrastructures throughout the century. Figs. S1-S4 show some examples of harmonized infrastructure layers.

### 2.4 Climate sensitivity of critical infrastructures (*S*)

A summary of the main vulnerabilities reported in the literature for the different sectors and hazards is presented in Tables S3-S6. Studies looking at the sensitivity of multi-sectorial critical infrastructures to climate hazards are lacking. Here, “sensitivity” refers to how much the asset or system is affected when exposed to a climate hazard. We therefore opted to construct a sensitivity matrix on the basis of a survey of experts and referred to the literature review to explain the channels through which the impacts are transmitted and to verify the robustness of the surveyed opinion. The web-based survey was set up using the secure European Commission tool EUSurvey (http://ec.europa.eu/eusurvey) and for each sector a sample of about 50 experts (out of 500 potential respondents) was collected from private companies, authors, and editorial boards of peer-reviewed journals in the field of climate change and sector-specific structural engineering. Experts anonymously assigned a degree of sensitivity (high, moderate, low, no) to infrastructures with respect to each climate hazard. We noted that respondents seem to sometimes confound (potentially unintentionally) exposure with sensitivity when they indicate the level of sensitivity. Evidence of such bias is the heterogeneity of answers from the survey about related infrastructures; for example, regional roads are estimated to be more sensitive to frost/snow/cold and floods than motorways and national roads. Exposure bias was removed to the extent possible based on literature about impacts and sensitivities, and by pooling responses for similar exposure assets per sector. The individual/personal representation of the overall impact of climate hazards and change was verified and shown to be very limited. We checked for individual bias by dropping the global representation of climate hazard impact within sectors, that is, removing the average of all answers for each respondent, and this check led to the same results. The modes of the resulting Likert distributions were considered to be representative of the sensitivity, and where there was low consensus among the experts and/or strong disagreement with reported impacts or sensitivities some adjustments were made based on the literature review. The sensitivity matrix (*S*) is shown in Table 1 and considered static over time.

**Table 1:** Sensitivity matrix. Sensitivity classes no (N), low (L), moderate (M) and high (H), followed by number of responses in the survey per class. Notes: a, answers about these assets were pooled per sector to remove exposure bias; b, sensitivity class changed based on impacts/sensitivities reported in literature (see Tables S3-S6); c, median of distribution taken instead of mode.

Sector	Infrastructure type	Heatwaves	Cold waves	Droughts	Wildfires	River and coastal floods	Windstorms
Energy	Coal power plants^a^	M (6,12,13,12)	L (14,21,7,0)	M (4,9,16,14)	L (9,15,8,9)	M (3,12,17,11)	M (3,22,18,1)^b^
	Gas power plants^a^	M (6,12,13,12)	L (14,21,7,0)	M (4,9,16,14)	L (9,15,8,9)	M (3,12,17,11)	M (3,22,18,1)^b^
	Oil power plants^a^	M (6,12,13,12)	L (14,21,7,0)	M (4,9,16,14)	L (9,15,8,9)	M (3,12,17,11)	M (3,22,18,1)^b^
	Nuclear power plants^a^	M (6,12,13,12)	L (14,21,7,0)	M (4,9,16,14)	L (9,15,8,9)	M (3,12,17,11)	M (3,22,18,1)^b^
	Biomass and geothermal power plants	M (7,8,16,12)	M (7,9,19,5)	H (5,9,6,23)	H (3,8,9,21)	M (5,13,15,9)	L (14,16,9,4)
	Hydro power plants	L (6,15,12,10)	M (5,17,15,4)^b^	H (2,1,7,34)	L (12,17,7,5)	M (3,7,18,14)	L (20,21,0,1)
	Solar power plants	N (21,13,6,3)	M (6,11,17,6)	N (26,12,2,2)	L (7,14,10,10)	L (10,21,8,4)	L (9,20,10,4)
	Wind power plants	N (24,11,5,2)	L (11,19,11,0)	N (31,9,3,0)	L (8,14,10,9)	L (7,23,9,5)	H (2,4,9,28)
	Electricity distribution/transmission	L (7,18,10,9)	M (3,9,22,9)	N (23,18,3,0)	H (3,7,11,21)	M (4,14,18,9)	H (4,7,14,19)
	Gas pipelines	N (23,14,3,4)	L (10,18,12,3)	N (28,15,1,0)	H (6,8,12,16)	L (6,17,10,11)	N (27,13,3,0)
**Transport**	Local roads^a^	M (9,21,19,2)^b^	M (2,8,22,20)	N (29,15,4,1)	M (4,15,24,7)	M (1,12,24,16)	L (4,22,15,8)
	Roads of national importance^a^	M (9,21,19,2)^b^	M (2,8,22,20)	N (29,15,4,1)	M (4,15,24,7)	M (1,12,24,16)	L (4,22,15,8)
	Motorways^a^	M (9,21,19,2)^b^	M (2,8,22,20)	N (29,15,4,1)	M (4,15,24,7)	M (1,12,24,16)	L (4,22,15,8)
	Railways	M (10,12,20,8)	M (1,10,27,13)	N (32,12,4,1)	M (4,12,29,5)	H (1,5,22,23)	L (10,19,14,7)
	Inland waterways	L (18,26,5,0)	M (2,12,26,10)	H (4,7,13,25)	L (8,26,5,0)	H (2,12,18,20)	M (6,22,18,4)^b^
	Ports	L (21,21,8,0)	M (4,18,23,5)	L (18,19,9,3)	L (21,21,8,0)	H (1,7,18,26)	M (7,17,17,10)
	Airports	L (10,23,14,2)	M (2,3,26,20)	N (30,18,1,0)	L (10,23,14,2)	M (6,16,23,8)	M (1,6,23,21)
**Industry**	Metal industry	L (7,19,9,2)	L (10,20,4,1)	L (10,12,10,5)	L (6,19,5,5)	M (2,8,13,12)	M (6,14,13,5)^b^
	Mineral industry	L (5,21,8,1)	L (10,17,6,0)	L (6,13,11,6)	L (6,20,5,4)	M (3,13,13,8)	M (5,14,13,4)^b^
	Chemical industry	L (6,18,10,2)	L (10,18,5,2)	L (10,11,9,7)	L (6,16,6,7)	M (3,12,12,8)	M (6,14,12,5)^b^
	Refineries	L (6,19,9,2)	L (10,18,5,1)	L (9,12,10,6)	L (6,18,6,6)	M (3,11,13,9)	M (6,14,12,5)^b^
	Water and waste treatment	M (5,20,15,6)^b^	M (7,18,19,2)	M (4,16,12,14)^c^	M (7,15,17,8)	H (3,3,19,22)	M (4,19,20,4)
**Social**	Education^a^	L (6,15,11,5)	L (5,16,10,4)	M (4,14,15,4)	M (5,10,12,10)	H (3,10,12,13)	M (7,12,11,6)^b^
	Health^a^	L (6,15,11,5)	L (5,16,10,4)	M (4,14,15,4)	M (5,10,12,10)	H (3,10,12,13)	M (7,12,11,6)^b^

### 2.5 Risk integration

For each infrastructure type, pan-European maps of potential risk levels (very high, high, moderate, low, very low, no) were constructed by multiplying hazard (*H*) and harmonized infrastructure layers (*E*). The resulting maps express how much infrastructure (in terms of sector-specific intensity value) in a particular cell is exposed to certain levels of risk, which are defined by the hazard intensity and the sensitivity of the infrastructure to the hazard, in accordance with a predefined risk matrix (Table 2). Only assets exposed to very high and high risk levels were considered to contribute to the impacts, assuming that no damage occurs to assets with no or low sensitivity to the hazard and from low-intensity hazard events. For the baseline period, the accumulated assets at very high and high risk levels for a specific hazard were linked to reported damage (measured in euros) for that hazard, derived from disaster databases.

**Table 2:** Risk matrix. Risk levels are expressed as a function of hazard intensity (classified according to return period *T_R_*) and sensitivity: no (N), very low (VL), low (L), moderate (M), high (H), very high (VH).

		Sensitivity			
		No (N)	Low (L)	Moderate (M)	High (H)
**Hazard intensity**	**Very high (*T_R_*** **≥** **100yr)**	N	M	H	VH
	**High (100yr>*T_R_*** **≥** **50yr)**	N	M	M	H
	**Moderate (50yr > *T_R_*** **≥** **20yr)**	N	L	M	M
	**Low (20yr > *T_R_*** **≥** **10yr)**	N	L	L	M
	**Very low (10yr > *T_R_*** **≥** **2yr)**	N	VL	L	L
	**No (*T_R_* < 2yr)**	N	N	N	N

To this end, more than 1100 disaster loss records for climate-related hazards that occurred in 1981–2010 were collected from the Emergency Events Database (EMDAT, http://www.emdat.be/database). Information for each disaster includes hazard type, country, year, and loss estimate. All data were converted into 2010 euros using the country’s Harmonized Index of Consumer Prices (HICP) derived from Eurostat. Information on disaster damage is available only at country level, without sectoral disaggregation. The baseline average annual damage derived from the individual records was distributed over specific sectors based on the national shares of the monetary value of sector-specific capital stock and gross value added (GVA) obtained from Eurostat and the sensitivity of sector infrastructures to the hazard under consideration. Sector-specific country damage was further disaggregated to nomenclature of territorial units for statistics level 2 (NUTS2) based on regional gross domestic product (GDP). From this data integration, we derive for each single hazard, infrastructure type, and NUTS2 region a relationship between assets exposed and damage, expressed as the ratio between the cumulated asset at very high/high risk levels and the reported damage. Such functions enable us to translate the intensity value of a given infrastructure at risk into the corresponding economic damage expressed in euros.

Future annual damage estimates were obtained by applying the asset exposed-damage relations to the projected changes of accumulated assets at high/very high risk levels, which are fully defined by the changes in hazard (*H*) as the sensitivity (*S*) and the spatial distribution of infrastructures (*E*) were assumed to be constant. This implies that we assume that the changes in the part of the frequency distribution that we consider to be linked with impacts (currently 50 years or less frequent for highly sensitive infrastructures and every 100 years or less frequent for infrastructures with medium sensitivity) are representative of the true changes in the frequency of damaging events. Baseline and future expected annual damage (EAD) values were calculated separately for all climate hazards, scenario periods, and climate experiments. This enables us to assess the climate model variability at single-hazard level. Multi-hazard damage was obtained by summing up single-hazard multi-model median EAD values (the hazards have only one climate representation in common; Table S1) under the assumption of static vulnerability (complete post-event recovery) and independent hazards (no hazard interrelations). As a conservative qualitative proxy of the propagation of the single-hazard climate uncertainty into the multi-hazard space, the uncertainty of the multi-hazard climate risk is expressed as the multi-model maximum and minimum of the impacts of each single hazard. Damage estimates have to be interpreted as structural damage to assets and losses due to production interruption according to the reported loss information in EMDAT. In order to build confidence in our methodological approach, we compare our damage estimates for river floods with those that Rojas et al. (2013) obtained by an independent approach using standard damage functions (Text S1).

### 2.6 Adaptation scenarios

In order to provide a first assessment of the additional investments needed to climate-proof infrastructures in different regions of Europe, the available literature on adaptation BCRs was surveyed. The studies reviewed (Table S7) provided a range of BCRs between 9 and 0.4, with an average value of 2.5. Following the approach described by Rojas et al. (2013), these BCR values have been used to provide indicative estimates (order of magnitude) of the potential cost of adaptation. Here, the direct benefits of adaptation equal the potential adverse impacts avoided, which are obtained as a difference in the damage to infrastructures between the future time period and the present (baseline). This represents an ideal scenario, as the theory and the evidence suggest that adaptation cannot generally overcome all climate change impacts and that some adaptation may not be physically possible or economically worthwhile (Parry et al., 2009). To derive indicative costs of adaptation, the literature-based average BCR value was combined with the projected benefits and expressed as a proportion of GDP. Furthermore, it is assumed that capital costs reflect 30% of the total adaptation cost over its lifetime and that they are incurred now, whereas operation and maintenance (O&M) costs (the remaining 70% of costs) are spread equally in time.

## 3 Results

### 3.1 Overall multi-hazard multi-sector risks

**Fig. 2:**
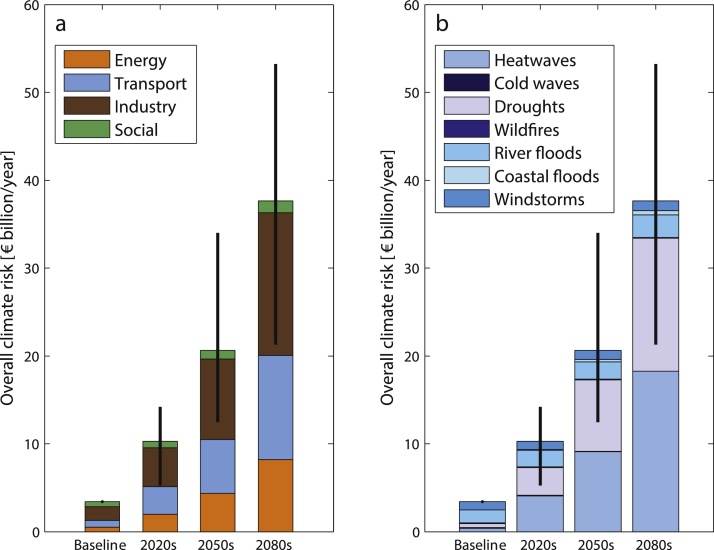
Overall climate hazard risk to critical infrastructures aggregated at European level (EU+) for each time period: a) distribution of damage by sector; b) distribution of damage over the seven hazards. For wind, projections of hazard are not available for 2020 s and 2050s; damage for these periods was obtained by linearly interpolating between the baseline and the 2080s. Whiskers reflect the inter-model climate variability.

The results show that Europe will face a continuous and ever sharper increase in multi-hazard multi-sector damage in the coming decades. The current overall EAD is €3.4 billion per year for EU+, but is projected to amount to approximately €9.3 billion (€5.2–14.2 billion uncertainty range), €19.6 billion (€12.5–34.0 billion) and €37.0 billion (€21.3–53.2 billion) per year by the 2020s, 2050s, and 2080s, respectively (Fig. 2), only as a result of the effects of climate change. The strongest rise in multi-hazard damage (Fig. 2a) is projected for the energy sector, for which the baseline EAD of €0.5 billion per year could rise to €1.8 billion (€1.1-2.8 billion), €4.2 billion (€3.0-6.7 billion) and €8.2 billion (€5.0-10.7 billion) per year (or increases in EAD of 394%, 860% and 1612%) by the 2020s, 2050s, and 2080s, respectively. A comparable trend can be observed for the transport sector, for which the baseline EAD of €0.8 billion per year is expected to reach €11.9 billion (€5.4–18.1 billion) per year (an increase of 1496%) by the end of this century. For industry, which faces the greatest damage among the sectors considered, EAD, currently €1.5 billion per year, is estimated to surpass €16.2 billion (€9.9–22.5 billion) per year by the 2080s, corresponding to a 10-fold increase. For the social sector, the rising trend in damage is less pronounced, but the current EAD of €0.6 billion per year could still more than double by the end of this century because of climate change.

Whereas current multi-sector hazard damage (Fig. 2b) relates mostly to river floods (44%) and windstorms (27%), the proportions of drought and heatwaves will rise strongly, to account for nearly 90% of climate hazard damage by the end of the century (vs 12% in the baseline period). This suggests that impacts of climate extremes could change not only in terms of the magnitude of damage, but also in their typologies. The relative contributions of wildfires and coastal floods to the overall projected damage are low, despite the strong increase in coastal flood damage that is projected for the coming century. The low contribution of present coastal flood damage may relate to the fact that EMDAT covers coastal impacts poorly, and coastal flood events can be reported under storms or floods. Therefore, part of the coastal flood damage is likely to be reflected in the inland flood and windstorm damage. Reported cold-related damage in Europe is marginal and could completely disappear with global warming.

### 3.2 Sector- and infrastructure-level risks

**Fig. 3:**
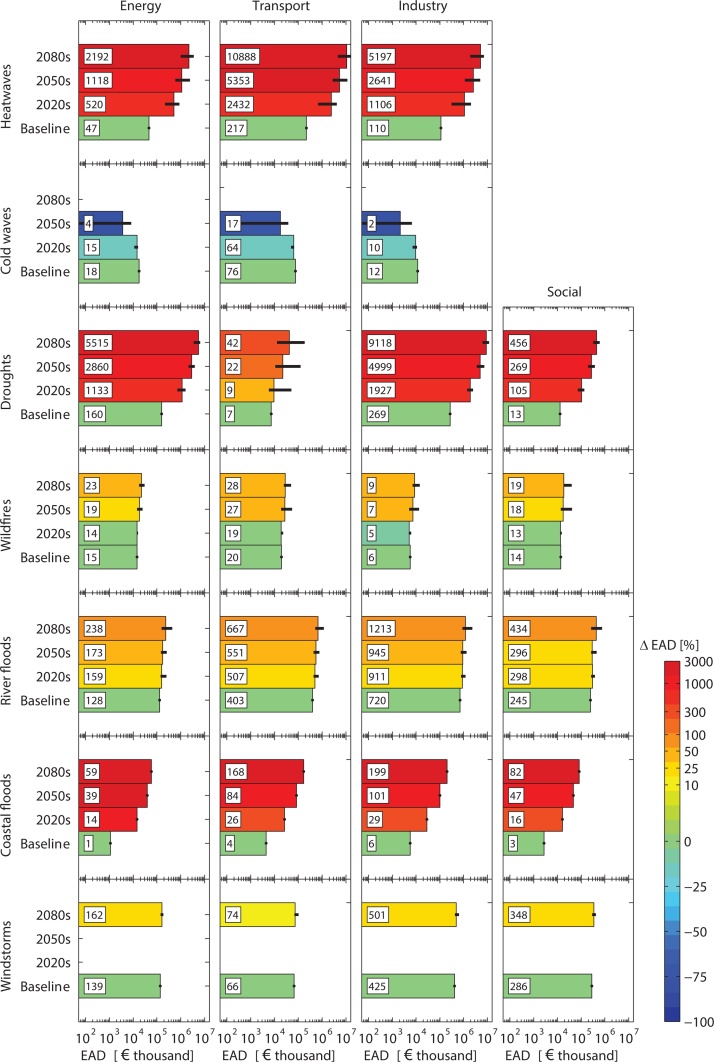
Expected annual damage (EAD) to critical infrastructures aggregated at European level (EU+) for each hazard, time period, and sector. Bar length indicates the ensemble median − also reported in numerical labels in millions. Whiskers reflect the inter-model climate variability (EAD for coastal floods has been produced for one climate configuration; Forzieri et al., 2016). Colors reflect the relative change in EAD with respect to the baseline.

**Fig. 4:**
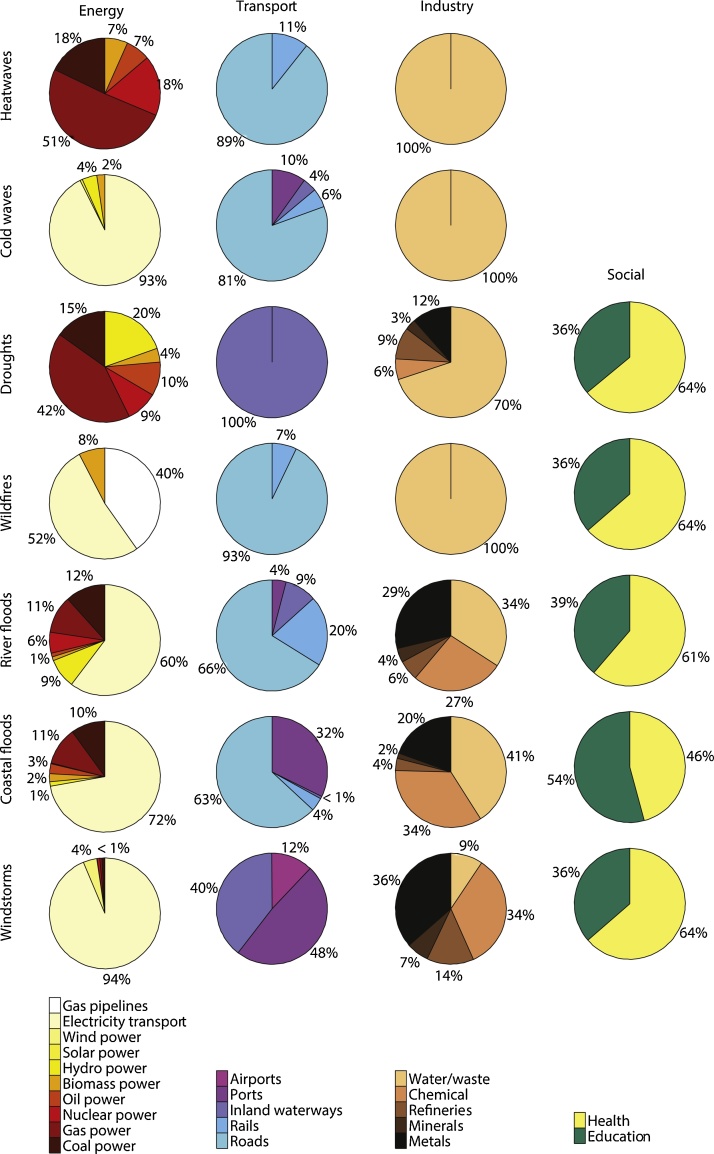
Distribution of hazard impacts over infrastructures types per sector, calculated over 2011–2100.

Hazard impacts and climate-induced dynamics therein (Fig. 3) vary among the different sectors and hazards (Fig. 4), with the actual damage and degree of change depending on sector-specific vulnerabilities (Tables S3-S6) to the different hazards and the rate and magnitude of change in the latter as a result of climate warming.

The largest rise in damage for the energy sector relates to energy production − fossil fuel, nuclear, and renewable − as a result of its sensitivity to droughts and heatwaves (e.g. decrease in cooling system efficiency of power plants due to higher water/air temperature). By the end of this century, drought and heat damage in Europe will comprise 67% and 27%, respectively, of all hazard impacts to the energy sector (now 31% and 9%, respectively). The other hazards mainly affect energy transport systems, and with time the hazard impacts show less distinct increases (wildfires, inland flooding, and windstorms), increase drastically in frequency but remain low in magnitude (coastal flooding) or decline sharply (cold waves).

For the transport sector, heatwaves will largely dominate future damage (92% of total hazard damage by 2080s), mainly by affecting roads and railways (e.g. buckling of rails, melting of asphalt). These modes of transport also suffer losses from inland ( > 50% current road and rail damage) and coastal flooding, which will moderately and drastically increase over time, respectively, as well as from cold waves (≈10% current road and rail damage) but with a strongly declining trend. Inland waterway transport will increasingly be affected by droughts (e.g. less navigation capacity due to low water levels in rivers), whereas windstorm damage to river navigation shows a slight increase. Sea level rise and increased storm surges will lead to strong increases in damage to ports in the coming century.

Floods and windstorms currently dominate hazard losses in the industry sector, mainly through structural damage to infrastructures, machinery, and equipment. Although flood and windstorm damage is on the rise, its contribution will be quickly outweighed by those of droughts and heatwaves in the coming decades. The impacts relate mostly to the degradation of water quality and a reduction of the decomposition rate of water and waste management systems, with corresponding higher costs for water and its treatment.

For the social sector, structural damage from flooding and windstorms will rise and remain important, whereas drought-induced subsidence damage could rise considerably. No damage is obtained for heatwaves and cold waves, as the sensitivity of education and health infrastructures to the hazards under consideration is low (Table 1).

### 3.3 Space-time variations of risks

**Fig. 5:**
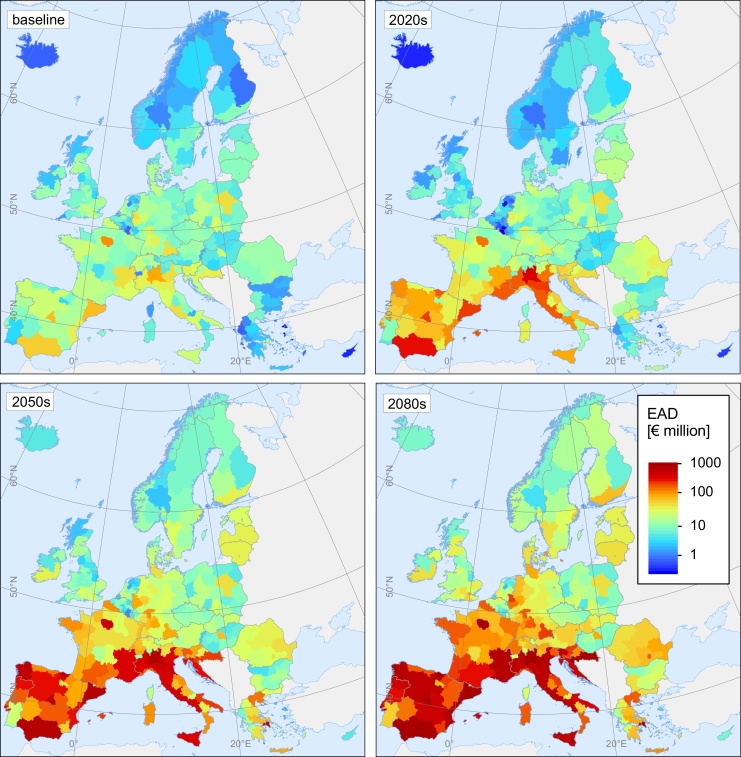
Spatial patterns of overall climate hazard risk to critical infrastructures in the different time periods.

The EU+ aggregated results mask the strong differences in impacts across Europe. Regional impacts depend on the spatial variations in the frequency of occurrence and magnitude of a (future) hazard, as well as on the spatial distribution of exposed assets and regional welfare. Detailed space-time variations in multi-hazard multi-sector impacts are visualized in Fig. 5 (maps of single-hazard single-sector EAD are shown in Figs. S5-S11). All regions of Europe are projected to experience a progressive increase in multi-hazard losses, but a noticeable pattern is the strong increase in damage load in southern Europe in the coming decades, with the most southerly regions progressively more prominently affected by future climate extremes than the rest of Europe. A large part of the north-south damage gradient relates to droughts, which will strongly intensify in southern parts of Europe and become less severe in northern regions (Forzieri et al., 2014). Given this, for sectors sensitive to this hazard, namely the energy and industry sector, drought-induced damage will strongly increase in the south and decrease in the north of Europe. Heatwaves also contribute to the north-south damage gradient, but to a lesser extent than droughts, as heatwave impacts are projected to rise significantly all over Europe yet more in the south. River and coastal floods will remain the most critical hazard in many floodplains and coastal stretches of western, central, and eastern Europe, including the British Isles, Poland, the Czech Republic, Bulgaria, Romania, and northern coastlines of the Iberian Peninsula.

For Europe as a whole, the damage by the seven hazards to the infrastructures under consideration, expressed as a proportion of the gross fixed capital formation (GFCF, a measure of the annual investments in fixed assets) at risk rises progressively from 0.12% at present to 1.37% by the end of this century (Table 3). The regional imbalance in impacts is reflected by the strong variations in the proportions of GFCF at risk within Europe. Whereas in northern Europe the damage from climate conditions by the end of this century represents less than 1% of annual investments, in southern European countries this damage corresponds to considerably higher proportions of annual fixed capital formation, especially for Italy (2.79%), Slovenia (3.01%), Portugal (4.29%), Spain (4.32%), Greece (4.43%) and Croatia (5.21%).

**Table 3:** Expected annual damage (EAD) and cost of adaptation (in 2010 constant euro prices or percentage of 2010 GFCF) for multi-hazard multi-sector analysis. Values for different time windows refer to results obtained by adding up single-hazard multi-model medians and reflect the EAD and adaptation costs assuming climate conditions of the time window imposed on present infrastructures. Note that for Cyprus, Malta and Iceland (coastal and river floods, and droughts) some hazards are not modelled, so no damage is included for these hazards in these countries.

Country	EAD (€ million)				EAD (% of GFCF)				Capital cost (€ million)			Capital cost (% of GFCF)			Annual O&M cost (€ million)		
	2000s	2020s	2050s	2080s	2000s	2020s	2050s	2080s	2020s	2050s	2080s	2020s	2050s	2080s	2020s	2050s	2080s
AT	72	134	224	485	0.11	0.21	0.35	0.76	223	771	2260	0.35	1.21	3.54	17.3	30	59
BE	58	77	136	237	0.07	0.10	0.17	0.30	70	350	994	0.09	0.44	1.25	5.4	13.6	26
BG	15	54	68	169	0.18	0.64	0.81	1.99	139	329	883	1.63	3.88	10.39	10.8	12.8	23
CH	136	261	530	910	0.14	0.26	0.53	0.91	449	1869	4655	0.45	1.86	4.64	35	73	121
CY	0	1	5	9	0.01	0.02	0.13	0.21	1	19	51	0.03	0.47	1.21	0.1	0.8	1.3
CZ	87	118	101	142	0.21	0.28	0.24	0.34	112	163	362	0.27	0.39	0.86	8.7	6.3	9.4
DE	579	1039	1788	2956	0.12	0.21	0.36	0.59	1657	6010	14,569	0.33	1.20	2.91	129	234	378
DK	66	124	187	291	0.15	0.28	0.42	0.66	210	646	1,458	0.48	1.47	3.31	16.3	25	38
EE	6	15	32	42	0.21	0.47	1.02	1.34	29	121	249	0.94	3.88	7.96	2.3	4.7	6.4
ES	394	2,270	5,344	10,759	0.16	0.91	2.15	4.32	6,753	24,572	61,885	2.71	9.87	24.85	525	956	1,604
FI	15	39	72	127	0.04	0.09	0.18	0.31	86	293	696	0.21	0.72	1.70	6.7	11.4	18
FR	432	1,368	2,865	5,378	0.10	0.31	0.65	1.22	3,372	12,131	29,937	0.76	2.75	6.79	262	472	776
GR	44	223	1,270	1,759	0.11	0.56	3.20	4.43	645	5,058	11,232	1.62	12.74	28.29	50	197	291
HR	21	55	163	499	0.22	0.57	1.70	5.21	122	635	2,357	1.28	6.63	24.62	10	25	61
HU	47	56	112	169	0.23	0.28	0.56	0.85	35	269	712	0.18	1.35	3.56	2.7	10.5	18
IE	13	21	49	55	0.04	0.07	0.17	0.19	29	159	311	0.10	0.54	1.05	2.3	6.2	8.1
IS	1	1	5	8	0.05	0.07	0.38	0.60	1	18	46	0.07	1.27	3.25	0.1	0.7	1.2
IT	460	2,617	4,901	8,939	0.14	0.82	1.53	2.79	7,768	23,756	54,282	2.43	7.42	16.96	604	924	1,407
LT	9	26	42	49	0.19	0.54	0.89	1.04	59	177	320	1.25	3.73	6.77	4.6	6.9	8.3
LU	6	8	11	20	0.08	0.12	0.15	0.28	9	26	77	0.12	0.36	1.07	0.7	1.0	2.0
LV	9	22	39	42	0.25	0.65	1.14	1.21	49	160	278	1.42	4.63	8.08	3.8	6.2	7.2
MT	0	10	9	10	0.01	0.73	0.63	0.74	36	68	105	2.58	4.82	7.44	2.8	2.6	2.7
NL	76	105	156	202	0.06	0.08	0.12	0.16	104	393	848	0.08	0.31	0.68	8.1	15.3	22
NO	19	31	69	113	0.03	0.05	0.10	0.17	41	221	558	0.06	0.33	0.84	3.2	8.6	14.5
PL	206	277	240	260	0.28	0.38	0.33	0.35	257	379	576	0.35	0.52	0.78	20.0	14.7	14.9
PT	48	376	900	1,583	0.13	1.02	2.44	4.29	1,180	4,249	9,776	3.20	11.50	26.47	92	165	253
RO	101	261	289	661	0.31	0.79	0.88	2.01	575	1,252	3,269	1.75	3.81	9.94	45	49	85
SE	52	87	180	239	0.06	0.11	0.22	0.29	126	587	1,257	0.15	0.71	1.53	9.8	23	33
SI	17	39	72	233	0.23	0.50	0.93	3.01	77	274	1,050	1.00	3.55	13.59	6	11	27
SK	19	27	82	208	0.13	0.18	0.55	1.39	29	258	938	0.19	1.73	6.29	2.2	10.0	24
UK	403	563	679	1,076	0.14	0.19	0.23	0.37	575	1,566	3,988	0.20	0.54	1.37	44.7	61	103
EU+	3,410	10,304	20,621	37,632	0.12	0.38	0.75	1.37	24,820	86,778	209,977	0.90	3.16	7.65	1,930	3,375	5,444

### 3.4 Cost of adapting infrastructures to climate change

Estimates of adaptation costs indicate that for EU+, taking into account only short-term projected changes in climate, costs to be incurred now would equal €25 billion, or 0.9% of EU+ 2010 GFCF, plus a yearly O&M cost of nearly €2 billion. This, however, would make infrastructures resilient to climate only up to 2040. The investments for adaptation required to face changes in climate in the medium term too (up to 2070) would amount to an upfront capital cost of €87 billion, or 3.16% of EU+ 2010 GFCF, and an annual O&M cost of €3.4 billion. To make infrastructures climate resilient up to the end of the century, capital costs could exceed €200 billion (about 7.65% of EU+ 2010 GFCF) and O&M costs could grow to €5.4 billion per year. These indicative numbers suggest that infrastructure projects with a long life span may require a substantial additional upfront investment to ensuring life-long resilience to climate hazards. Adaptation costs will not fall equally across Europe. Countries in southern Europe that will be exposed to higher risk levels could potentially have to direct a significant proportion of their investments in fixed capital to abating the future impacts from climate hazards on critical infrastructures (Table 3).

It is stressed that these indicative costs are subject to many factors, such as the shape of the marginal cost curve for enhancing resilience against increasing extreme hazard intensity, the balance between soft and hard options, and the balance of capital and O&M costs, among others. Nonetheless, they suggest that adaptation of critical infrastructures could be a cost-effective strategy, but that costs to be incurred could be considerable for several countries in Europe.

### 3.5 Main limitations and knowledge gaps in methodological and data aspects

While the reasonable agreement between our risk estimates and those reported by Rojas et al. (2013) corroborates the overall hazard-exposure-sensitivity integration framework proposed here (Text S1 and Fig. S12), a series of potential limitations should be carefully considered.

Our multi-hazard risk framework is built on the propagation of baseline damage to future scenarios according to variations in the frequency of extreme events and the spatial distribution of exposed assets. Hence, any deviations of the reported damage from the true impacts are inherently translated into our damage estimates. At present, our understanding of long-term climate risks is limited by the lack of in-depth knowledge on the impacts of climate hazards, due to the absence of harmonized loss data recording. Baseline damage for this study is retrieved from the EMDAT loss databases. While it is one of the most comprehensive sources of reported impacts of climate-related disasters, the recorded losses most likely deviate from the true numbers (Felbermayr and Gröschl, 2014, Gall et al., 2009). As the data that populate the database originate from different sources and are collected by multiple actors, the loss figures should be viewed bearing in mind their potential biases.

The national recorded hazard damage retrieved from the disaster database has been disaggregated across sectors/infrastructures and NUTS2 regions based on the regional societal and economic structure as represented by Eurostat statistics and the sensitivities to the specific hazards derived from the survey and the literature. The assumptions beyond the proposed disaggregation of losses represent potential sources of uncertainty resulting from the incomplete knowledge about the true sector-specific impacts and their spatialization (Meyer et al., 2013). Although reasonable assumptions have been formulated, such epistemic uncertainties are difficult to assess.

In this study, we assume independent hazards and static vulnerability. However, hazards may induce or reinforce other hazards, and they may overlap spatially and temporally, as observed by Forzieri et al. (2016), influencing not only the overall hazard level, but also the vulnerability of elements at risk through possible hazard interrelations or cascade effects (Kappes et al., 2012). The scarcity of observational relations linking variations in multi-hazard impacts on vulnerability does not allow a reliable integration of such effects in large-scale predictive systems.

Furthermore, vulnerability as derived from the survey does not account for different degrees of interconnectivity, technological heterogeneity, and the life span of infrastructures, which may influence susceptibility to climate extremes. However, we emphasize that the aim of the analysis was to derive general sensitivities for types/classes of infrastructures across a great territorial diversity with a wide variety of socio-economic settings and physical boundary conditions in Europe, ensuring comparability in the multi-hazard and multi-sector context.

In our adaptation scenario, we consider a uniform BCR to derive a first assessment of the additional investments needed to climate-proof infrastructures in different regions of Europe. However, adaptation measures are very diverse and usually take place at the local level, with diverse regulatory, legal, and governance settings. These determine the type of measures chosen and level of investments as well as the scale at which the measures are implemented and the associating costing framework (Berkhout et al., 2015, Bouwer et al., 2013). Such local-scale information is not available at pan-European level and therefore is not considered in this study.

Climate-change impact uncertainties are quantified in this study solely in terms of the spread induced by the climate-model projections, and do not account for all the sources of uncertainty detailed above. We recognize that the impact-model spread of our damage projections can be comparable to, or even larger than, the spread introduced by the different climate models considered (Piontek et al., 2014).

Impacts of extremes may go far beyond the physical assets themselves. Wider economic, social, and environmental effects depend on the institutional and economic environments, especially on the upward and downward sides of the production chain and thus on the dependency networks of critical infrastructures, which are complex systems. Interdependencies, cascading effects, and the risk of failures were not explicitly modelled in this study for lack of metrics or models that satisfactorily capture these aspects for highly interconnected infrastructures, especially for an application at the continental scale. Rather, it has been assumed that such wider consequences are implicit in the reported damage. Disaster risk databases, however, are typically poor at reflecting indirect, inter-sectorial effects and intangible damage. Hence, figures reported herein may potentially underestimate the full impacts of climate extremes on the sectors investigated.

## 4 Conclusions

This study has aimed to estimate the regional impacts across Europe of the seven most damaging climate hazards on the present stock of critical infrastructures. To this end, we integrated at pan-European scale state-of-the-art multi-hazard modelling, detailed exposure information, present knowledge on vulnerability derived from literature and expert views, and recorded disaster losses. Despite the breadth and depth of the analysis, estimates are subject to many caveats and uncertainties that reflect the present gaps in knowledge. The main challenge for further research in this area lies in the quantification of vulnerabilities of various types of infrastructures/sectors to the different climate hazards. Loss data systems in the EU and other parts of the world are fragmented and inconsistent (Mysiak et al., 2016), and an important step to improving our understanding of infrastructure/sector vulnerability would be to introduce standardized reporting and sharing practices of data related to disaster damage and losses. Recent actions, such as the agreement on the global Sendai Indicators (United Nations, 2016) and alignment of national loss databases that comply with them, as well as the guidance document for EU Member States on Recording and Sharing Disaster Damage and Loss Data (De Groeve et al., 2015), aim to pave the way for improved disaster loss data collection and should be further encouraged and supported.

Notwithstanding that our estimates are subject to uncertainty, they do highlight some important issues. The predicted upsurge in climate hazard damage to infrastructures in Europe in the coming decades underpin the recent efforts of the EU to augment the profile of climate change in its budget and policies (European Commission, 2011, European Council, 2013, European Parliament and European Council, 2013, Hjerp et al., 2012). The distribution of economic costs in space and among sectors provides an indication of the regions and sectors that may require substantial efforts to make present and planned critical infrastructures resilient to the future climate. It emphasizes the importance of mainstreaming of climate change adaptation in a wide range of EU policies and funding instruments. Given the high level of interconnectedness of infrastructures, a cross-sectorial consideration of strategies for climate change adaptation and resilience should be encouraged.
