# Supplementary material for: Escalating impacts of climate extremes on critical infrastructures in Europe
Source: Glob Environ Change. 2018 Jan;48:97–107. doi: 10.1016/j.gloenvcha.2017.11.007 (PMC5872142; doi:10.1016/j.gloenvcha.2017.11.007)
Supplement: Supplementary file 1 [file mmc1.docx]

**Escalating impacts of climate extremes on critical infrastructures in Europe**

**Supplementary Information**

**Text S1.** Cross-comparison analysis

**Table S1.** Ensemble of GCM-RCM configurations used to derive the climate hazards in the period 1981–2100

**Table S2.** List of infrastructures

**Table S3.** Overview of vulnerability of energy assets to climate hazards, and impacts of hazards on assets, reported in literature

**Table S4.** Overview of vulnerability of transport assets to climate hazards, and impacts of hazards on assets, reported in literature

**Table S5.** Overview of vulnerability of industry assets to climate hazards, and impacts of hazards on assets, reported in literature

**Table S6.** Overview of vulnerability of social (education and health) assets to climate hazards, and impacts of hazards on assets, reported in literature

**Table S7.** Benefit-to-cost ratios (BCR) of adaptation measures reported in literature

**Figure S1.** Example of harmonized energy infrastructure layers

**Figure S2.** Example of harmonized transport infrastructure layers

**Figure S3.** Example of harmonized industry infrastructure layers

**Figure S4.** Example of harmonized social infrastructure layers

**Figure S5.** Expected annual damage due to heatwaves

**Figure S6.** Expected annual damage due to cold waves

**Figure S7.** Expected annual damage due to droughts

**Figure S8.** Expected annual damage due to wildfires

**Figure S9.** Expected annual damage due to river floods

**Figure S10.** Expected annual damage due to costal floods

**Figure S11.** Expected annual damage due to windstorms

**Figure S12.** Cross-comparison analysis of expected annual damage due to river floods

**References**

**Text S1. Cross-comparison analysis**

In order to build confidence in our methodological approach, we compare our damage estimates for river floods with those reported by Rojas et al. (2013; hereafter RA2013). Those authors use a well-established approach that translates simulated flood inundation depth into economic damage using empirically derived flood depth-damage functions. We point out that the cross-comparison analysis proposed should not be viewed as a validation, as we compare results of two modelling exercises, but the intention is to quantify the degree of agreement of our risk assessment with previous studies and understand possible divergences due to methodological aspects. For this purpose, the flood risk estimates reported in RA2013 represent an ideal comparative dataset because they are consistent with the hazard projections of our study (same ENSEMBLES climate model configurations, LISFLOOD hydrological modeling, and extreme value analysis). The use of common modelling features helps to identify possible differences in damage estimates related only to the risk integration methodological aspects. Furthermore, focusing the cross-comparison analysis on flood-related risks, we minimize the potential of initial methodological bias in the assessments compared with other hazards: flood damage in EMDAT utilized in our method is notoriously more extensively recorded than other hazards, whereas damage functions such as those utilized in RA2013 represent a well-consolidated approach to flood risk estimation for which the scientific community has attained greater confidence with respect to the risks connected to other climate extremes.

Before performing the cross-comparison analysis, some pre-processing was needed to improve the consistency of the experiment. Country-level expected annual damage (EAD) obtained for RA2013, which refer to the impacts on the overall economy, were scaled to account only for the portions of the total (reported) flood damage that relate to critical infrastructures as considered in our work. Furthermore, we disentangled the scaled risk to separate the damage related to changes in society/economy and climate, and only the latter is considered, consistent with our estimates generated under the hypothesis of no socio-economic changes. These risk estimates have been compared with our multi-sector flood-related EAD at country level and for each time period, and are shown in Figure S12. Reliability coefficients, expressed in terms of coefficient of determination (*R*^2^), Pearson correlation (*ρ*) and percent bias (pbias) are computed for each time slice and shown in colored labels according to the legend.

We found a general good agreement between the two methodologies (*R*^2^ and *ρ* up to 0.46 and 0.68, respectively) with a progressively increasing match for longer term future scenarios. RA2013 systematically reports more damage than our estimates (pbias ranging between -29.03% and -60%), with more evident discrepancies for the Baltic countries, Finland, Hungary, and the Netherlands.

Our baseline EAD estimates coincide with damage recorded in EMDAT. Thus, the initial -29.03% pbias could be imputable to an overestimation by RA2013 of the historically observed flood damage or – conversely – to an underestimation of the actual flood damage in EMDAT. The bias further relates to the fact that energy, industry, social, and transport infrastructures are grouped under the “residential” land cover class in RA2013, without distinguishing differences in vulnerability among sector-specific infrastructures. As residential buildings have usually higher capital stock than many infrastructures considered here (e.g. roads, rails), this can lead to a general overestimation by RA2013 of the risks of critical infrastructures.

The pbias tends to increase in magnitude (with negative sign) quite sharply in 2020s (≈70% increase) and then more moderately for subsequent time periods (≈10%). This suggests that other factors in addition to those discussed above contribute to the discrepancies between the two modelling results. We argue that damage functions applied independently at pixel level and expressed in terms of water depth by RA2013 can lead to larger damage because they reproduce the impacts using three dimensions (two-dimensional horizontal space plus water depth). Future flood impacts are quantified by RA2013 not only in terms of changes in flooded areas and frequency of occurrence, as expressed in our indicator of *H* (see Method, main text), but also accounting for variations in inundation depth.

While recognizing that this cross-comparison analysis is neither fully consistent, in terms of damage reported and sectors investigated, nor comprehensive, as it explores only one of the seven hazards considered in our multi-hazard assessment, we believe that the reasonable agreement between the two approaches corroborates the overall framework proposed here. We believe that our risk estimates can probably provide better information about future risk levels for those critical infrastructures for which no specific damage functions have been developed.

| **Driving GCM** | **RCM** | **Acronyms** | **Heatwaves** | **Cold waves** | **Droughts** | **Wildfires** | **River floods** | **Windstorms** |
| --- | --- | --- | --- | --- | --- | --- | --- | --- |
| HadCM3Q16  ARPEGE  ARPEGE  BCM  ECHAM5-r3  HadCM3Q0  ECHAM5-r3  HadCM3Q0  ECHAM5-r3  BCM  ECHAM5-r3  HadCM3Q3 | RCA3.0  ALADIN-RM5.1  HIRHAM5  HIRHAM5  HIRHAM5  CLM  RACMO2  HadRM3Q0  REMO  RCA3.0  RCA3.0  RCA3.0 | C4I-RCA-HadCM3  CNRM-ALADIN-ARPEGE  DMI-HIRHAM5-ARPEGE  DMI-HIRHAM5-BCM  DMI-HIRHAM5-ECHAM5  ETHZ-CLM-HadCM3  KNMI-RACMO2-ECHAM5  METO-HadRM3-HadCM3  MPI-REMO-ECHAM5  SMHI-RCA-BCM  SMHI-RCA-ECHAM5  SMHI-RCA-HadCM3 | X  X  X  X  X | X  X  X  X  X | X  X  X  X  X  X  X  X  X  X  X  X | X  X  X  X  X | X  X  X  X  X  X  X  X  X  X  X  X | X  X  X  X |

**Table S1 |** Ensemble of GCM-RCM configurations used to derive the climate hazards in the period 1981–2100 (Forzieri et al., 2016).

| **Sector** | **Sub-sector** | **Infrastructure type** | **Data structure** | **Weights (*w*) used for harmonization**  $Y_{j,i}=X_{j,Z}\cdot\left( {w_{j,i}}/{\sum_{i\in Z} w_{j,i}} \right)$ | **Main source(s)** | **Reference date** |
| --- | --- | --- | --- | --- | --- | --- |
| **Transport** | Roads | Local roads | Vector (lines) | *w* = *L*·*C*·*P* (*L* = road network length; *C* ={5 = ‘motorways’, 3 = ‘national roads’, 2 = ‘local roads’}; *P* = number of residents within a radius of 20 km (Batista e Silva et al., 2013)) | Open Street Map | 2014 |
|  |  | Roads of national importance |  |  |  |  |
|  |  | Motorways |  |  |  |  |
|  | Other modes | Railways | Vector (lines) | *w* = *L*·*F* (*L* = rail or inland waterway network length; *F* = average freight flow (Ibánez-Rivas, 2010)) |  |  |
|  |  | Inland waterways |  |  | GISCO + UNECE | 2013 |
|  |  | Ports | Vector (points) | - | CORINE Land Cover + GISCO | 2006 |
|  |  | Airports |  | - |  |  |
| **Energy** | Non-renewable energy production | Coal power plants | Vector (points) | *w* = *I* (*I* = installed capacity) | PLATTS | 2013 |
|  |  | Gas power plants |  |  |  |  |
|  |  | Oil power plants |  |  |  |  |
|  |  | Nuclear power plants |  |  |  |  |
|  | Renewable energy production | Biomass and geothermal power plants | Vector (points) |  |  |  |
|  |  | Hydro power plants |  |  |  |  |
|  |  | Solar power plants |  |  |  |  |
|  |  | Wind power plants |  |  |  |  |
|  | Energy transport | Electricity distribution/transmission | Vector (lines) | *w* = *L*·*V* (*L* = electrical grid length; *V* = voltage) |  |  |
|  |  | Gas pipelines |  | *w* = *L*·*D* (*L* = gas pipeline length; *D* = pipeline diameter) |  |  |
| **Industry** | Heavy industries | Metal industry | Vector (points) | *w* = *N* (*N* = number of facilities) | EPRTR v7 | 2013 |
|  |  | Mineral industry |  |  |  |  |
|  |  | Chemical industry |  |  |  |  |
|  |  | Refineries |  |  | Global Energy Observatory | 2010 |
|  | Water/waste treatment | Water and waste treatment | Vector (points) |  | EPRTR v7 | 2013 |
| **Social** | Education | Education infrastructure | Vector (points) | *w* = *P*/*N* (*P* = number of residents within a radius of 20 km (Batista e Silva et al., 2013), *N* = number of facilities) | Open Street Map | 2014 |
|  | Health | Health infrastructure |  |  |  |  |

**Table S2 |** List of infrastructures considered in this study, sources used, reference dates, and local attributes employed for the harmonization procedure. The following notation is used in the equation: $Y_{j,i}$ is the intensity of infrastructure *j* in pixel *i*; $X_{j,Z}$ is the total intensity (or volume) of infrastructure *j* in country *Z*, as reported by Eurostat at national level; $w_{j,i}$ are the weights of infrastructure *j* in pixel *i* expressed as a function of local attributes.

|  | **Heatwaves** | **Cold waves** | **Droughts** | **Wildfires** | **River and coastal floods** | **Windstorms** |
| --- | --- | --- | --- | --- | --- | --- |
| **Energy** | Reduction of structural integrity due to melting permafrost (Cruz and Krausmann, 2013; Ebinger, 2011; Paskal, C, 2010)  Deterioration of power systems (Bompard et al., 2013)  Increased resistance on the power lines and electric power transformer failures (Schaeffer et al., 2012)  Decrease in power plant efficiency due to higher water/air temperature required for cooling systems (Chandramowli and Felder, 2014; Linnerud et al., 2011; Mideksa and Kallbekken, 2010; Paskal, C, 2010; Rübbelke and Vögele, 2011; Sieber, 2013; van Vliet et al., 2012; Vliet et al., 2013)  Reduction in biofuel sources (Moiseyev et al., 2011)  Coal stockpiles may be subject to spontaneous combustion (Sieber, 2013)  Reduction in solar and photovoltaic efficiency (Patt et al., 2013)  Expansion in gas and oil pipelines (Sieber, 2013) | Structural damage due to increased ice and snow loads on overhead distribution lines (Bompard et al., 2013)  Increased corrosion of energy systems (Sieber, 2013)  Reduction in hydropower generation due to water freezing (Bompard et al., 2013)  Reduction in biofuel sources (Schaeffer et al., 2012)  Reduction in solar and photovoltaic efficiency (Patt et al., 2013)  Contraction in gas/oil pipelines (Sieber, 2013)  Freezing of coal to ground (Sieber, 2013) | Reduction of structural integrity due to drought-induced subsidence (Cruz and Krausmann, 2013; Ebinger, 2011; Paskal, C, 2010)  Deterioration of power systems caused by overexploitation of water pumping (Rübbelke and Vögele, 2011)  Decrease in power plant efficiency due to higher water temperature and lower water volumes required for cooling systems (Bompard et al., 2013; Chandramowli and Felder, 2014; Ebinger, 2011; Linnerud et al., 2011; Mideksa and Kallbekken, 2010; Mima and Criqui, 2015; Paskal, C, 2010; Patt et al., 2013; Rübbelke and Vögele, 2011; Sieber, 2013; van Vliet et al., 2012; Vliet et al., 2013)  Deterioration of cooling systems due to excessive biological growth clogging water intakes (Cruz and Krausmann, 2013)  Reduction in biofuel sources (Moiseyev et al., 2011; Schaeffer et al., 2012)  Reduction in hydropower potential due to reduced water volumes (Lehner et al., 2006; Mima and Criqui, 2015; Schaeffer et al., 2012; van Vliet et al., 2012; Vliet et al., 2013) | Direct damage to power system equipment, pipelines, and electricity transmission lines from bushfires (Bompard et al., 2013; Mitchell, 2013; Sathaye et al., 2013) | Structural damage to energy production sites and transport networks due to direct impacts of flows, reduced soil stability, and induced mass movements (soil erosion, landslide, siltation) (Bompard et al., 2013; Brown et al., 2013; Chandramowli and Felder, 2014; Cruz and Krausmann, 2013; Ebinger, 2011; Schaeffer et al., 2012; Sieber, 2013)  Damage to power system equipment due to debris and pollution in cooling water flows (Cruz and Krausmann, 2013; Sieber, 2013)  Short-circuiting and power failure on electrical systems (Brown et al., 2013)  Disabling of protection equipment by corrosion and pitting (Cruz and Krausmann, 2013)  Reduction in biofuel sources (Ebinger, 2011)  Reduction in hydropower production due to increased silting of sediment into reservoirs (Chandramowli and Felder, 2014) | Structural damage to power system equipment and storage tanks due to wind pressure or debris impact (Bompard et al., 2013; Chandramowli and Felder, 2014; Ebinger, 2011; Pryor et al., 2005; Pryor and Barthelmie, 2010; Sieber, 2013)  Disruption of electricity lines due to falling trees (Schaeffer et al., 2012)  Short-circuiting triggering possible fires especially with storage of liquid flammable hydrocarbons (Bompard et al., 2013; Sieber, 2013)  Reduction in biofuel sources (Bompard et al., 2013; Schaeffer et al., 2012)  Stresses on wind turbines (Mima and Criqui, 2015)  Deposition and abrasive effects of wind-blown sand and dust on solar energy plants, reduction of power output, and need for cleaning (Patt et al., 2013) |

**Table S3 |** Overview of vulnerability of energy assets to climate hazards, and impacts of hazards on assets, reported in literature.

|  | **Heatwaves** | **Cold waves** | **Droughts** | **Wildfires** | **River and coastal floods** | **Windstorms** |
| --- | --- | --- | --- | --- | --- | --- |
| **Transport** | Buckling of roads, railways, and bridges due to thermal expansion; structural material degradation; melting of asphalt and increased rutting and softening of pavement, signaling problems (Chinowsky et al., 2013; Ciscar JC, Feyen L, Soria A, Lavalle C, Raes F, Perry M, Nemry F, Demirel H, Rozsai M, Dosio A, Donatelli M, Srivastava A, Fumagalli D, Niemeyer S, Shrestha S, Ciaian P, Himics M, Van Doorslaer B, Barrios S, Ibáñez N, Forzieri G, Rojas R, Bianchi A, Dowling P, Camia A, Libertà G, San Miguel J, de Rigo D, Caudullo G, Barredo J-I, Paci D, Pycroft J, Saveyn B, Van Regemorter D, Revesz T, Vandyck T, Vrontisi Z, Baranzelli C, Vandecasteele I, Batista e Silva F, Ibarreta D, 2014; Dobney et al., 2009; Hooper and Chapman, 2012; Jaroszweski et al., 2010; Leviäkangas, P et al., 2011; Palin et al., 2013; Vajda et al., 2013)  Increased numbers of tire blow-outs (Jaroszweski et al., 2010)  Effects of denser air on aviation and reduced engine combustion efficiency (Hooper and Chapman, 2012; Jaroszweski et al., 2010)  Impacts of overheating on infrastructure equipment: reduction in reliability of the electronic and electric components (e.g. rail rolling stock equipment) over their lifetime (Ciscar JC, Feyen L, Soria A, Lavalle C, Raes F, Perry M, Nemry F, Demirel H, Rozsai M, Dosio A, Donatelli M, Srivastava A, Fumagalli D, Niemeyer S, Shrestha S, Ciaian P, Himics M, Van Doorslaer B, Barrios S, Ibáñez N, Forzieri G, Rojas R, Bianchi A, Dowling P, Camia A, Libertà G, San Miguel J, de Rigo D, Caudullo G, Barredo J-I, Paci D, Pycroft J, Saveyn B, Van Regemorter D, Revesz T, Vandyck T, Vrontisi Z, Baranzelli C, Vandecasteele I, Batista e Silva F, Ibarreta D, 2014; Palin et al., 2013)  Slope instabilities due to the thawing of permafrost (Hooper and Chapman, 2012; Jaroszweski et al., 2010; Leviäkangas, P et al., 2011) | Icing of aircraft wings, vessels, decks, riggings, and docks, and disruption of transport functioning (Doll et al., 2014; Leviäkangas, P et al., 2011; Pejovic et al., 2009)  Disruption of boat traffic due to thick river ice cover (Schweighofer, 2013; Vajda et al., 2013)  Supply cable sag or tensional failure (rail) (Leviäkangas, P et al., 2011) | Reduced navigability of rivers and channels due to low-level streamflows (Hooper and Chapman, 2012; Jonkeren et al., 2007, 2013; Koetse and Rietveld, 2009; Leviäkangas, P et al., 2011; Middelkoop et al., 2001; Schweighofer, 2013) | Disruption of roads and interruption of traffic (Leviäkangas, P et al., 2011) | Reduction in structural integrity of surface and subgrade material due to wave action and induced mass movements (erosion, landslide, subsidence) (Ciscar JC, Feyen L, Soria A, Lavalle C, Raes F, Perry M, Nemry F, Demirel H, Rozsai M, Dosio A, Donatelli M, Srivastava A, Fumagalli D, Niemeyer S, Shrestha S, Ciaian P, Himics M, Van Doorslaer B, Barrios S, Ibáñez N, Forzieri G, Rojas R, Bianchi A, Dowling P, Camia A, Libertà G, San Miguel J, de Rigo D, Caudullo G, Barredo J-I, Paci D, Pycroft J, Saveyn B, Van Regemorter D, Revesz T, Vandyck T, Vrontisi Z, Baranzelli C, Vandecasteele I, Batista e Silva F, Ibarreta D, 2014; Jaroszweski et al., 2010; Koetse and Rietveld, 2009; Leviäkangas, P et al., 2011; Suarez et al., 2005)  Scour on bridges and embankments, track and rail lineside equipment failure (Ciscar JC, Feyen L, Soria A, Lavalle C, Raes F, Perry M, Nemry F, Demirel H, Rozsai M, Dosio A, Donatelli M, Srivastava A, Fumagalli D, Niemeyer S, Shrestha S, Ciaian P, Himics M, Van Doorslaer B, Barrios S, Ibáñez N, Forzieri G, Rojas R, Bianchi A, Dowling P, Camia A, Libertà G, San Miguel J, de Rigo D, Caudullo G, Barredo J-I, Paci D, Pycroft J, Saveyn B, Van Regemorter D, Revesz T, Vandyck T, Vrontisi Z, Baranzelli C, Vandecasteele I, Batista e Silva F, Ibarreta D, 2014; Leviäkangas, P et al., 2011; Suarez et al., 2005; Wright et al., 2012)  Deterioration of infrastructures that lack a fouling-resistant design against salt water (Molarius et al., 2013)  Disruption of transport vehicles and facilities (Hallegatte et al., 2010; Koetse and Rietveld, 2009; Leviäkangas, P et al., 2011; Vajda et al., 2013)  Reduced navigability of rivers and channels due to lower clearance under waterway bridges and increased sedimentation (Koetse and Rietveld, 2009; Leviäkangas, P et al., 2011; Schweighofer, 2013) | Structural damage to transport facilities due to wind pressure or debris impact (Hooper and Chapman, 2012; Leviäkangas, P et al., 2011)  Short-circuiting along electrical cables (Leviäkangas, P et al., 2011)  Obstruction of roads and rails due to fallen vegetation (Leviäkangas, P et al., 2011)  Disruption of air and boat traffic due to high turbulences (Leviäkangas, P et al., 2011; Schweighofer, 2013)  Danger that piled shipping containers may tip over (Leviäkangas, P et al., 2011; Vajda et al., 2013) |

**Table S4 |** Overview of vulnerability of transport assets to climate hazards, and impacts of hazards on assets, reported in literature

|  | **Heatwaves** | **Cold waves** | **Droughts** | **Wildfires** | **River and coastal floods** | **Windstorms** |
| --- | --- | --- | --- | --- | --- | --- |
| **Industry** | Increased methane production and potential leachate escape (Bebb, J and Kersey, J, 2003)  Higher treatment costs due to increased distribution of vermin and pests (Bebb, J and Kersey, J, 2003)  Reduction in decomposition rate leading to lower operability and productivity (Bebb, J and Kersey, J, 2003)  Degradation of water quality and increased cost for treatment (Delpla et al., 2009; Tang et al., 2013) | Vulnerability of water pipes to frost/icing (Bebb, J and Kersey, J, 2003) | Increased risk of subsidence (Bebb, J and Kersey, J, 2003)  Water quality degradation, reduction in drinkable water, and increase in treatment costs (Delpla et al., 2009; Middelkoop et al., 2001; Tang et al., 2013; Whitehead et al., 2009)  Reduction in decomposition rate leading to lower operability and productivity (Bebb, J and Kersey, J, 2003; Delpla et al., 2009) | Forest fires affecting the viability of mining operations and potentially increasing operating, transportation, and decommissioning costs (Ford et al., 2009)  Direct impact on production sites and sector facilities (Nielsen-Pincus et al., 2014) | Structural damage to industrial sites due to direct impacts of flows, reduced soil stability, and induced mass movements (soil erosion, landslide, siltation) (Bebb, J and Kersey, J, 2003; Cozzani et al., 2010; Krausmann et al., 2011)  Increased disruption of supporting infrastructure, e.g. weighbridges and gas and leachate collection systems (Bebb, J and Kersey, J, 2003)  Increasing degradation of water quality and increased cost for water treatment (e.g. due to release of pollutants into the drainage and sewer system when brownfields are exposed to overflows) (Delpla et al., 2009; Langeveld et al., 2013; Major et al., 2014; Neumann et al., 2014)  Increased intrusion of salty water, , water quality degradation, interruption to pumping of wells, increasing cost of water treatment, and reduction of water available for industrial purposes (Cozzani et al., 2010; Krausmann et al., 2011; Major et al., 2014) | Structural damage to industrial system equipment due to wind pressure or debris impact (Bebb, J and Kersey, J, 2003; Krausmann et al., 2011) |

**Table S5 |** Overview of vulnerability of industry assets to climate hazards, and impacts of hazards on assets, reported in literature.

|  | **Heatwaves** | **Cold** | **Droughts** | **Wildfires** | **River and coastal floods** | **Windstorms** |
| --- | --- | --- | --- | --- | --- | --- |
| **Social** |  |  | Structural damage due to drought-induced subsidence and permafrost thawing (Corti et al., 2009, 2011)  Higher dependence of people on water availability and quality (Pita et al., 2013) |  | Structural damage to industrial sites due to direct impacts of overflows, reduced soil stability, and induced mass movements (soil erosion, landslide, siltation) (Hallegatte et al., 2010; Oven et al., 2012; Radovic et al., 2012)  Reduction in operational services (Carmichael et al., 2012; Hallegatte and Corfee-Morlot, 2011) | Structural damage to education and health structures and facilities due to wind pressure or debris impact (Hallegatte et al., 2010; Pita et al., 2013; Stewart et al., 2011) |

**Table S6 |** Overview of vulnerability of social (education and health) assets to climate hazards, and impacts of hazards on assets, reported in literature.

|  | **Adaptation measure** | **BCR (average and range)** | **Region** | **Reference** |
| --- | --- | --- | --- | --- |
| **Infrastructures** | Prevention of storm damage to buildings | 2.7 (1.3-4.8) | Germany | (Tröltzsch et al., 2012) |
|  | Local structural protection | 1.7 | Austria | (Holub and Fuchs, 2008) |
| **Industry** | Awareness raising for companies | 5.3 (1.0-9.7) | EU | (Hjerp et al., 2012) |
| **Energy** | Adaptation of electricity grids | 5.1 | EU26, without Malta | (Hjerp et al., 2012) |
|  | High-efficiency ventilation | 1.8 | EU26, without Malta | (Hjerp et al., 2012) |
| **Transport** | Improved road pavement materials and design standards | 3 | Germany and Austria | (Doll et al., 2014) |
|  | Adapting tracks (e.g., railways, tram lines) to higher temperatures | 2 (0.34-9) | EU | (Hjerp et al., 2012) |
|  | Adapting roads to higher temperatures | 0.4 (0.2-0.9) | EU | (Hjerp et al., 2012) |
|  | Adapting roads to increase in precipitation | 0.5 (0.1-1.9) | EU | (Hjerp et al., 2012) |
|  | Transport and spatial planning: general protection measures | 1.3 | EU | (Doll et al., 2011) |
|  | Transport and spatial planning: network redesign | 1.2 | EU | (Doll et al., 2011) |
|  | Infrastructure measures: incentives and information | 2.4 | EU | (Doll et al., 2011) |
|  | Infrastructure measures: supervision and maintenance | 1.2 | EU | (Doll et al., 2011) |
|  | Infrastructure measures: investments | 1.5 | EU | (Doll et al., 2011) |
|  | Vehicle technologies: detection and communication | 1.2 | EU | (Doll et al., 2011) |
|  | Vehicle technologies: vehicle engineering | 1.9 | EU | (Doll et al., 2011) |
|  | Vehicle technologies: maintenance | 1 | EU | (Doll et al., 2011) |
|  | Service operations: raising preparedness | 1.4 | EU | (Doll et al., 2011) |
|  | Service operations: co-operation strategies | 3.8 | EU | (Doll et al., 2011) |
|  | Service operations: system redesign | 0.7 | EU | (Doll et al., 2011) |
| **Cross-cutting** | Building dykes and beach nourishment | 2.5 | Germany | (Tröltzsch et al., 2012) |
|  | Storm retention reservoirs | 3.5 (0.5-9.4) | EU | (Hjerp et al., 2012) |
|  | Action plan on flood defense for River Rhine | 3.4 | River Rhine (Germany) | (Petrascheck, 2003) |
|  | Flood and coastal risk management in England | 7.5 (4-11) | UK | (EA, 2009) |
|  | Flood risk management plan in Belgium | 4.1 | Scheldt Estuary (Belgium) | (Broekx et al., 2011) |
|  | Early warning for flash floods | 9 | Germany | (Schröter et al., 2008) |
|  | Groins | 3.2 (1.6-4) | Greece | (Kontogianni et al., 2014) |
|  | Beach nourishment | 2.1 (0.4-3.8) | Greece | (Kontogianni et al., 2014) |
|  | Revetments and geotextiles | 3.7 (3.3-3.9) | Greece | (Kontogianni et al., 2014) |
|  | Bulkheads | 3.3 (2.4-3.9) | Greece | (Kontogianni et al., 2014) |

**Table S7 |** Benefit-to-cost ratios (BCRs) of adaptation measures reported in literature

**
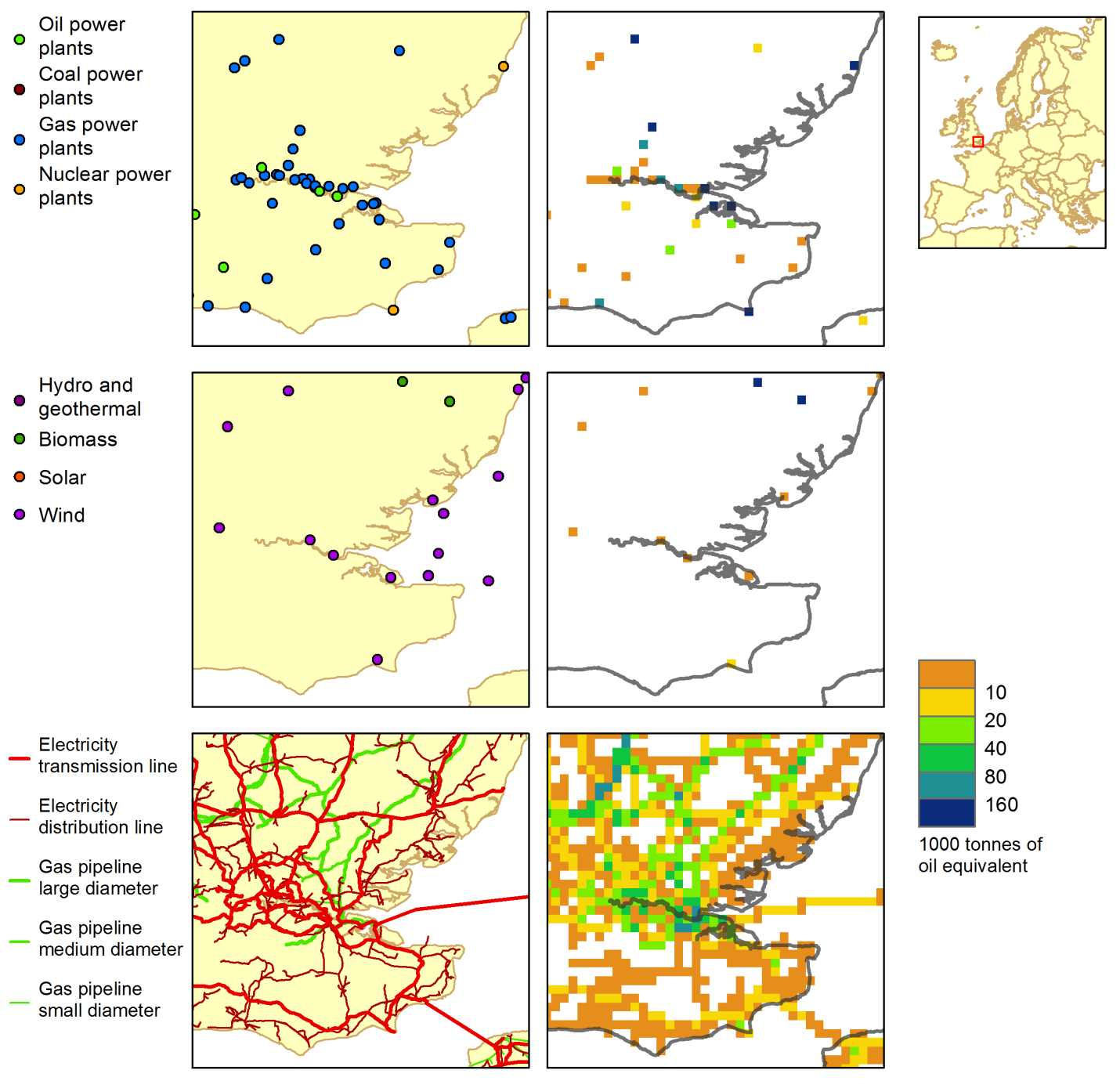
**

**Figure S1 |** Example of harmonized energy infrastructure layers.

**
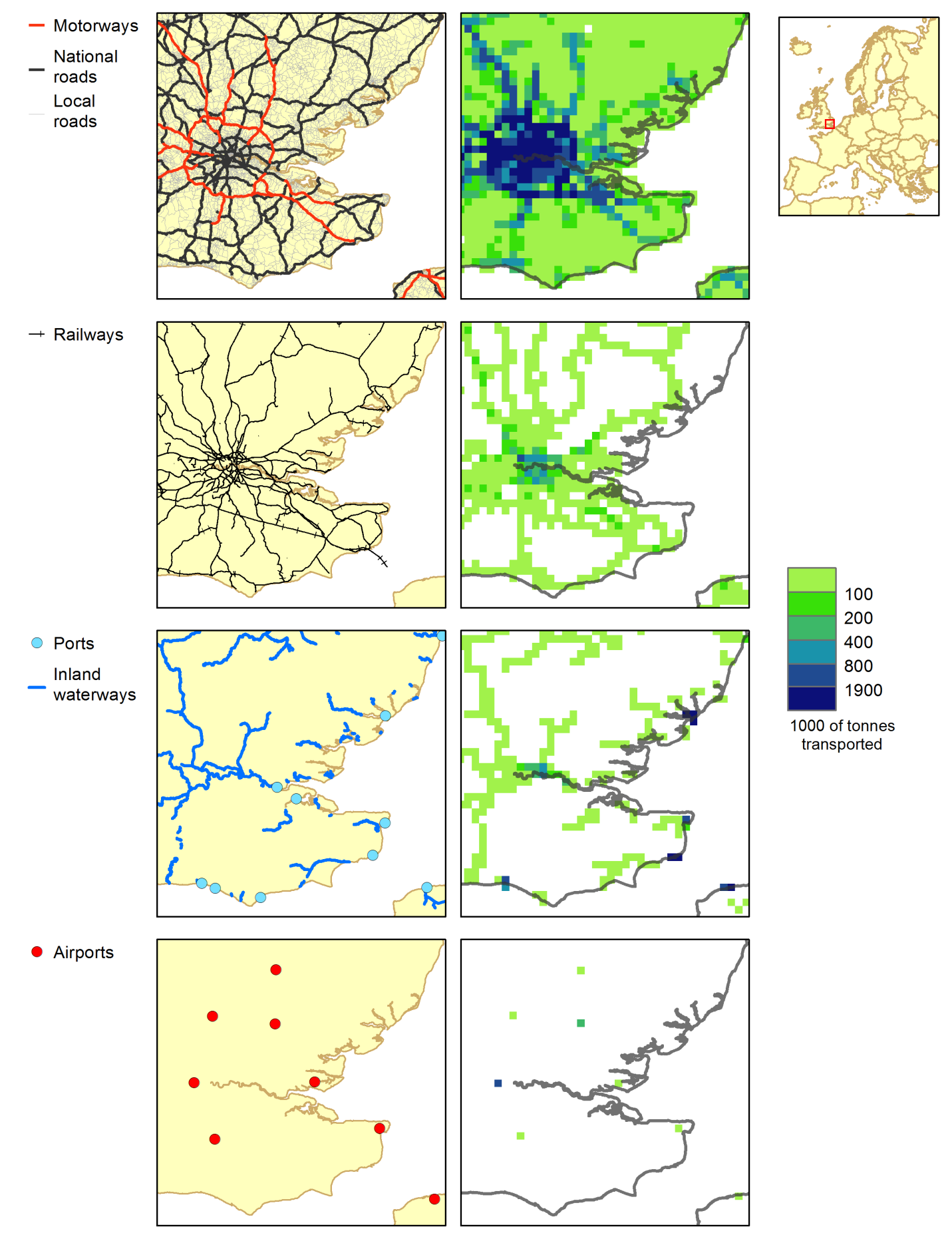
**

**Figure S2 |** Example of harmonized transport infrastructure layers.

**
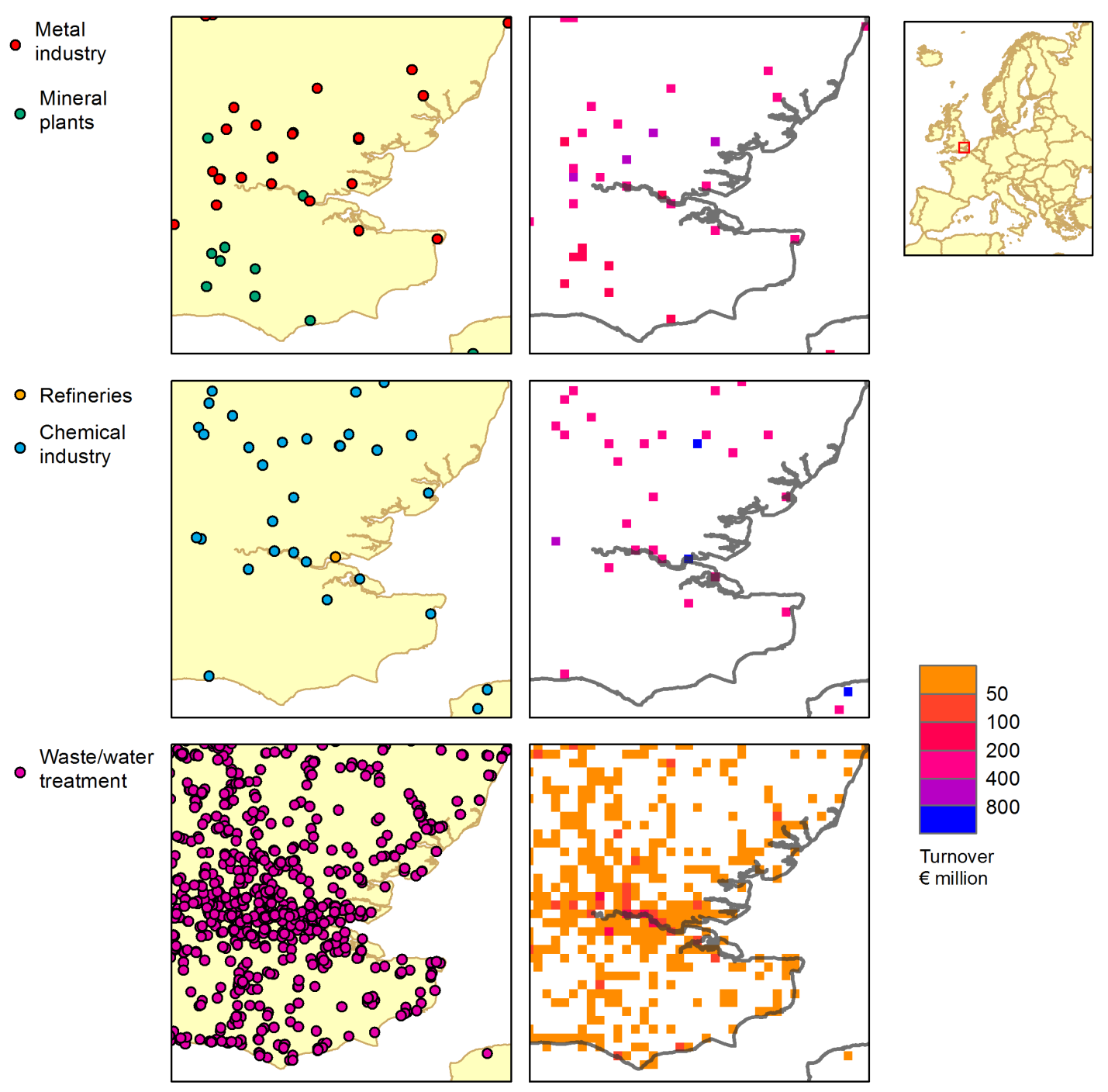
**

**Figure S3 |** Example of harmonized industry infrastructure layers.

**
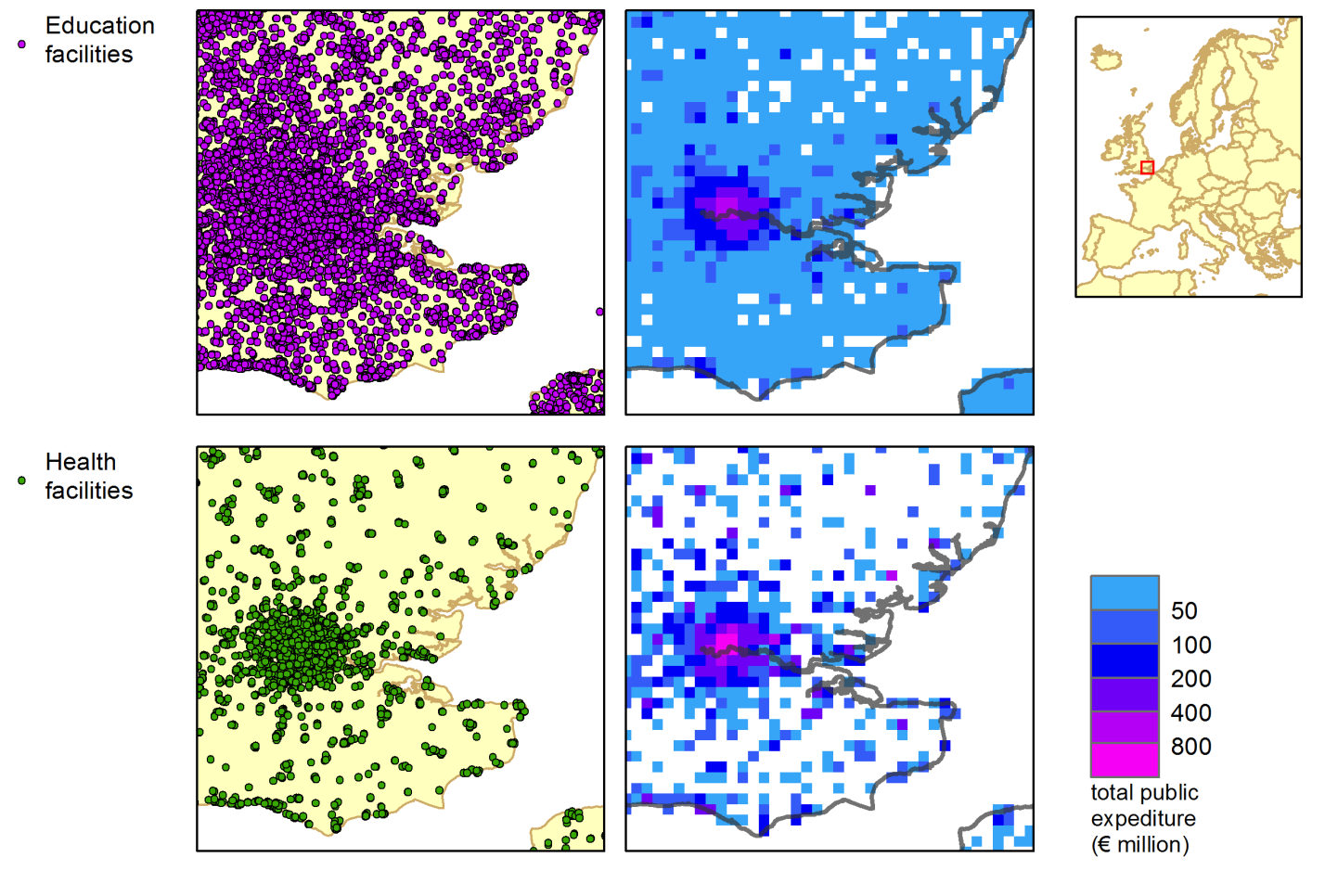
**

**Figure S4 |** Example of harmonized social infrastructure layers.


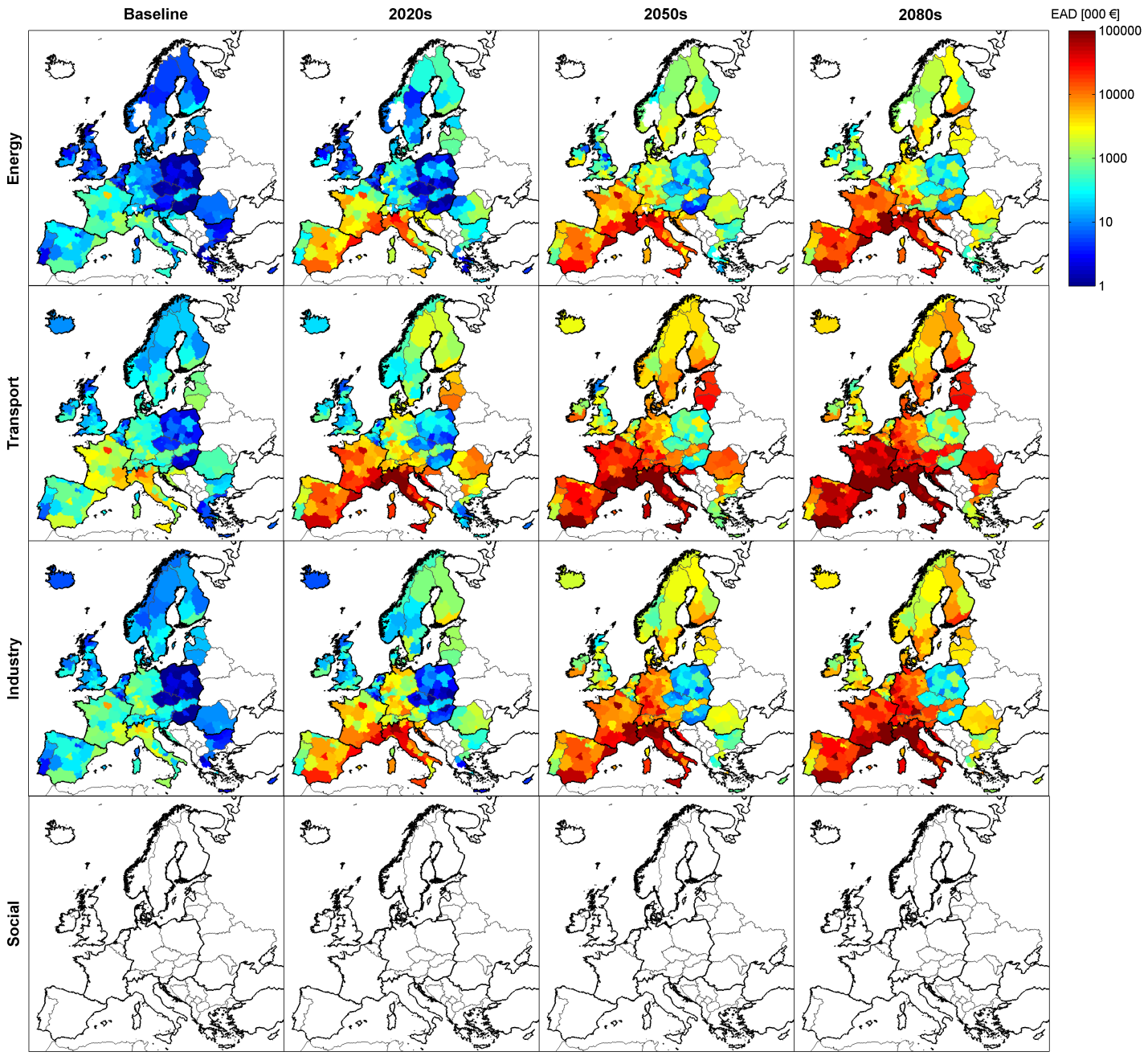


**Figure S5 |** Expected annual damage due to heatwaves.

**
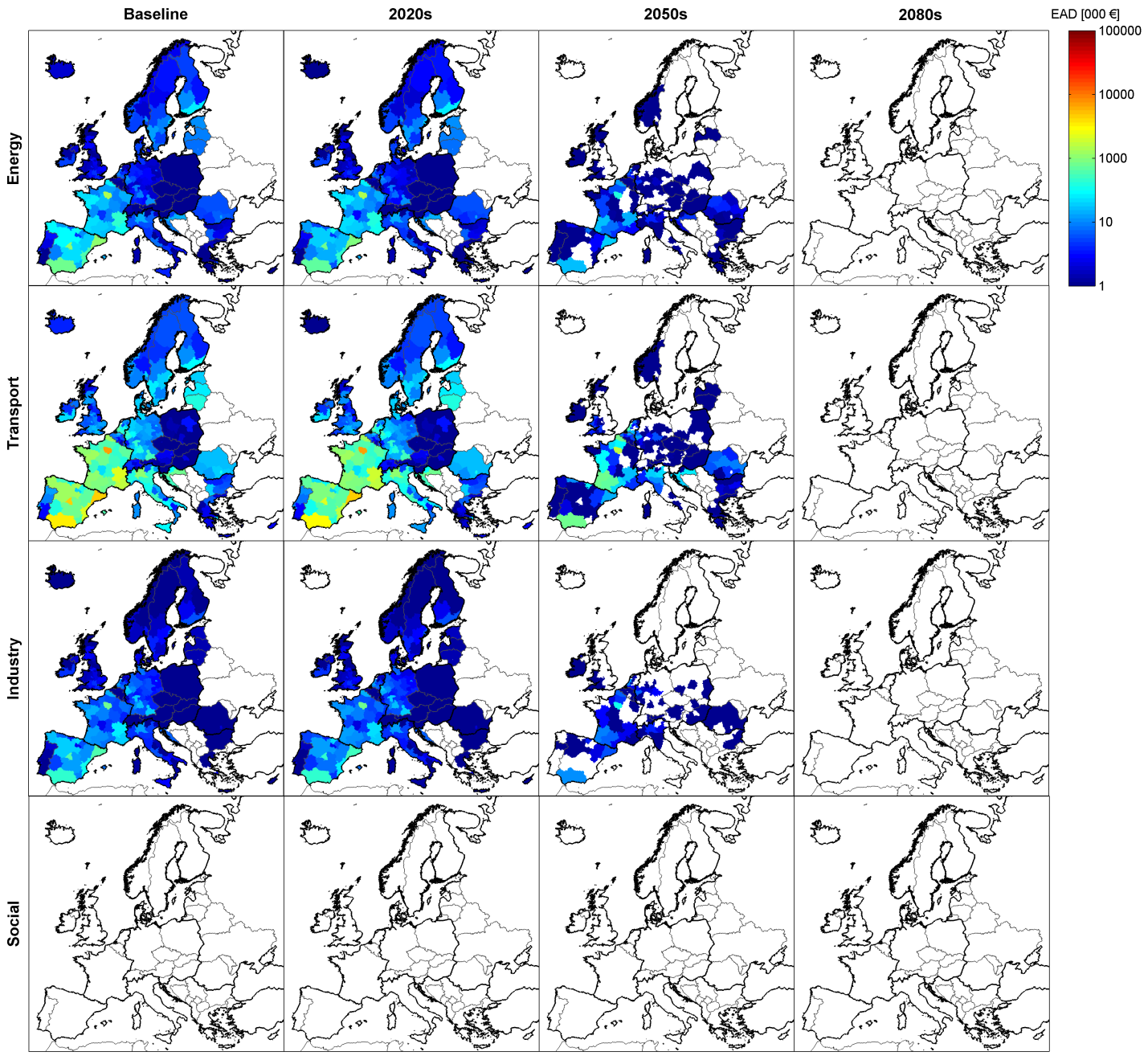
**

**Figure S6 |** Expected annual damage due to cold waves.


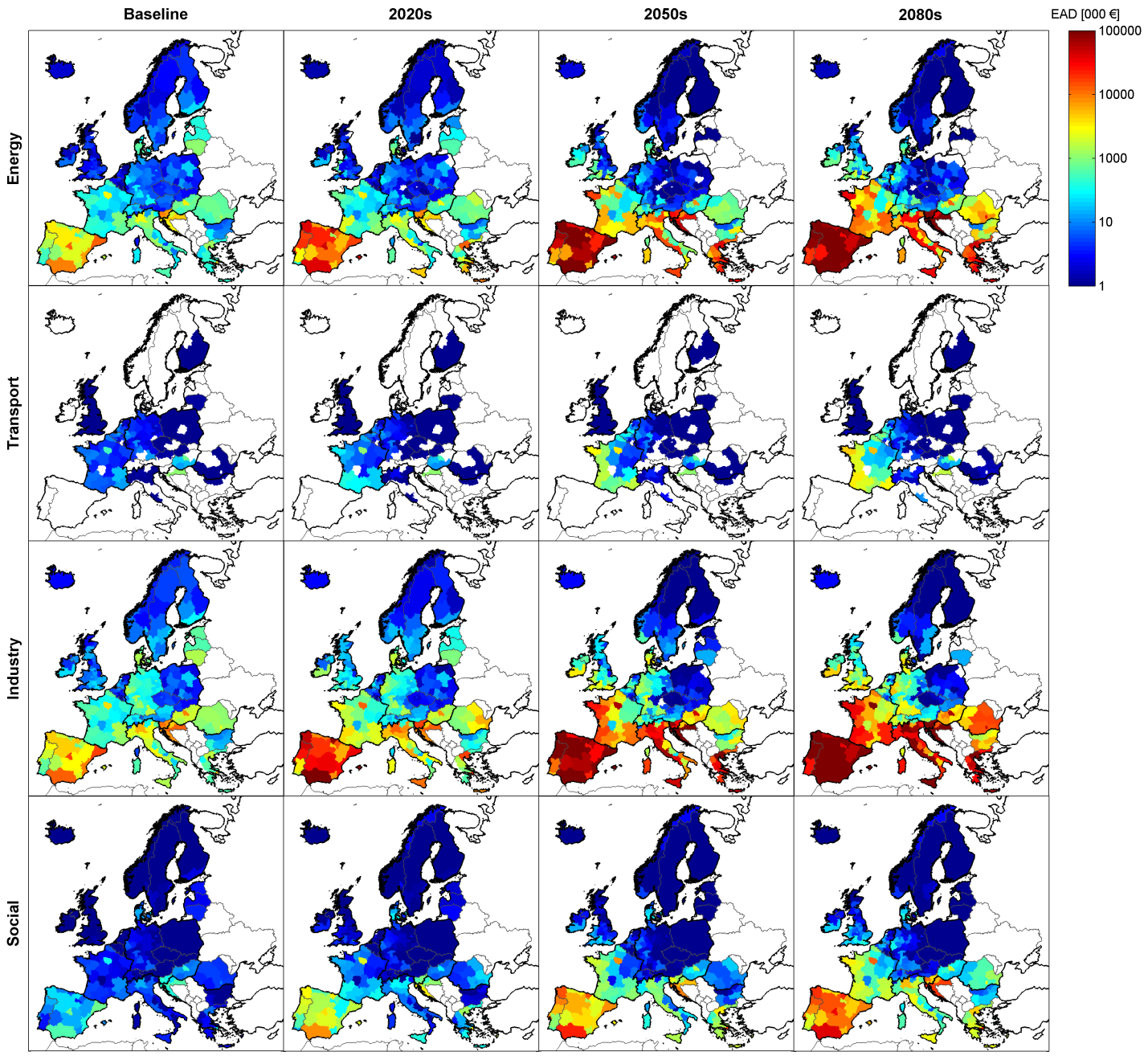


**Figure S7 |** Expected annual damage due to droughts. **
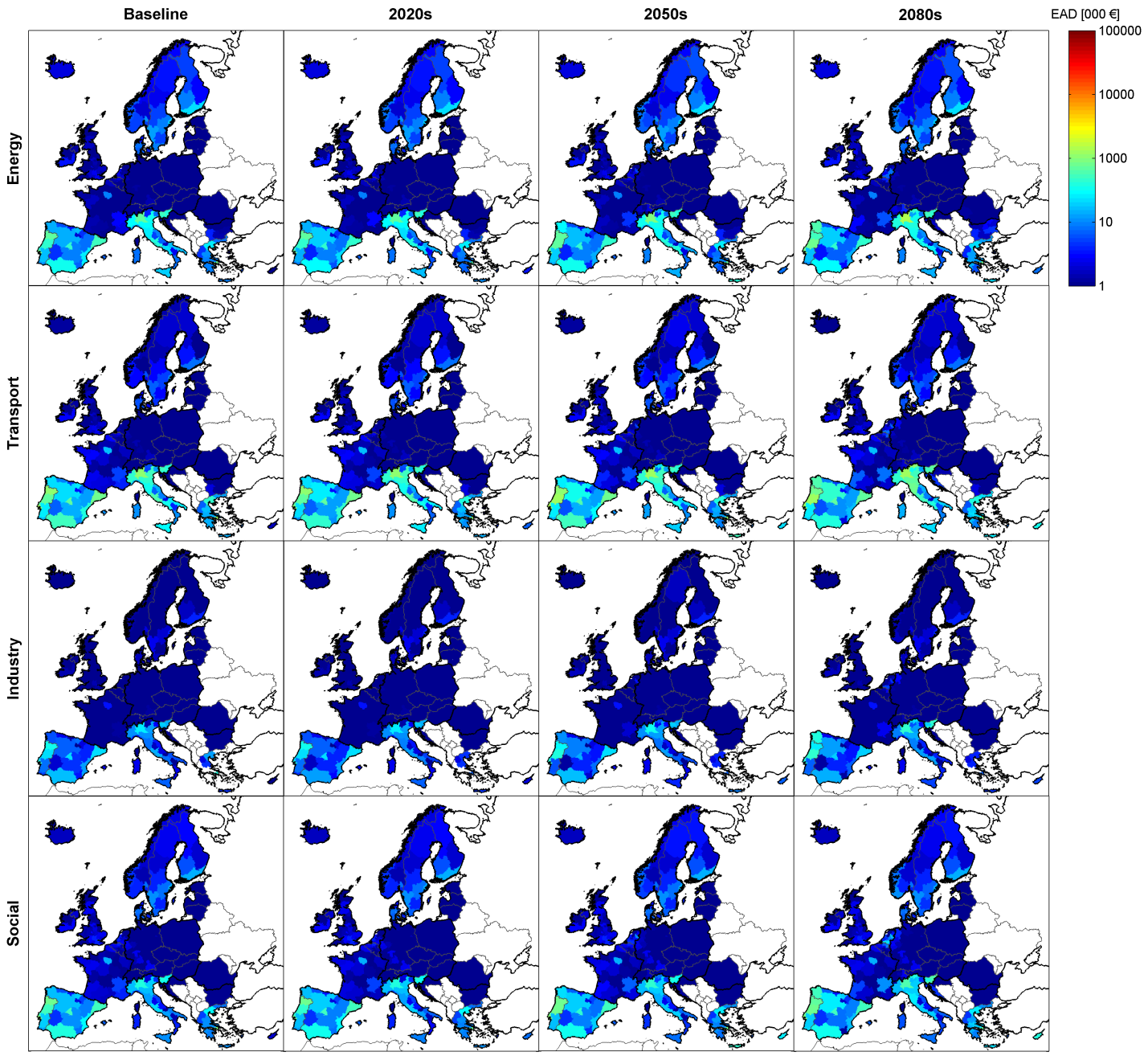
**

**Figure S8 |** Expected annual damage due to wildfires.

**
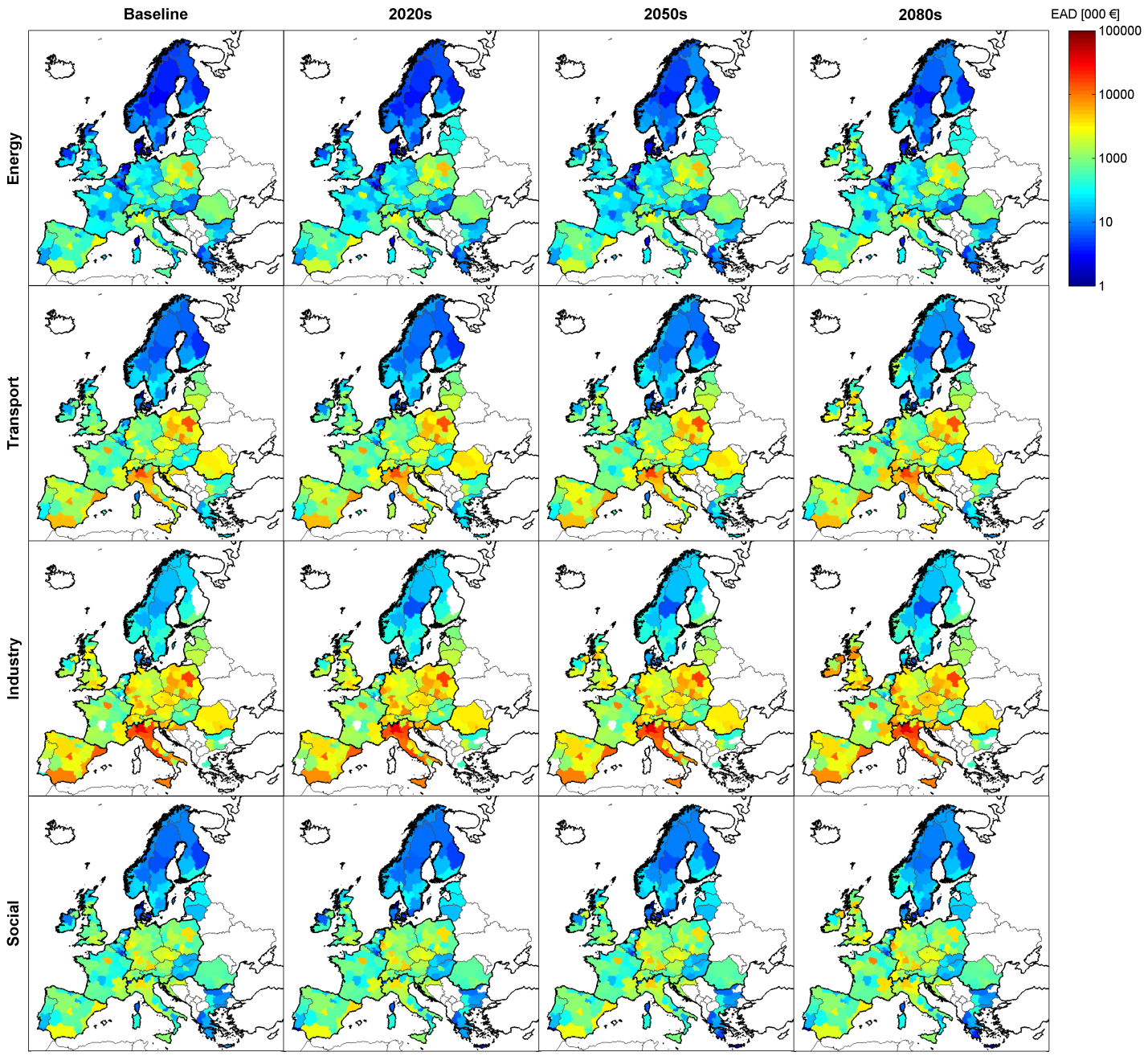
**

**Figure S9 |** Expected annual damage due to river floods.

**
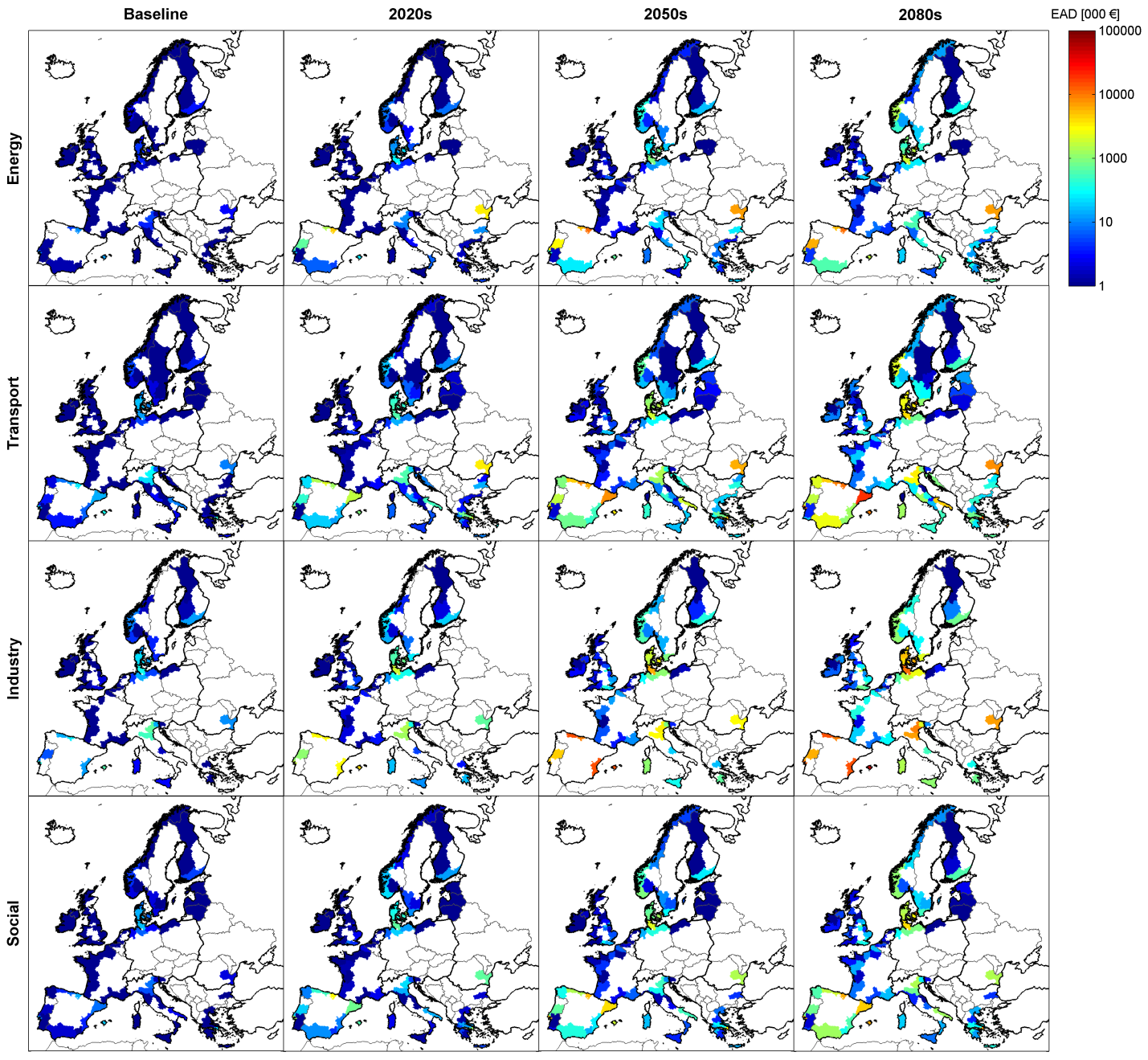
**

**Figure S10 |** Expected annual damage due to costal floods.

**
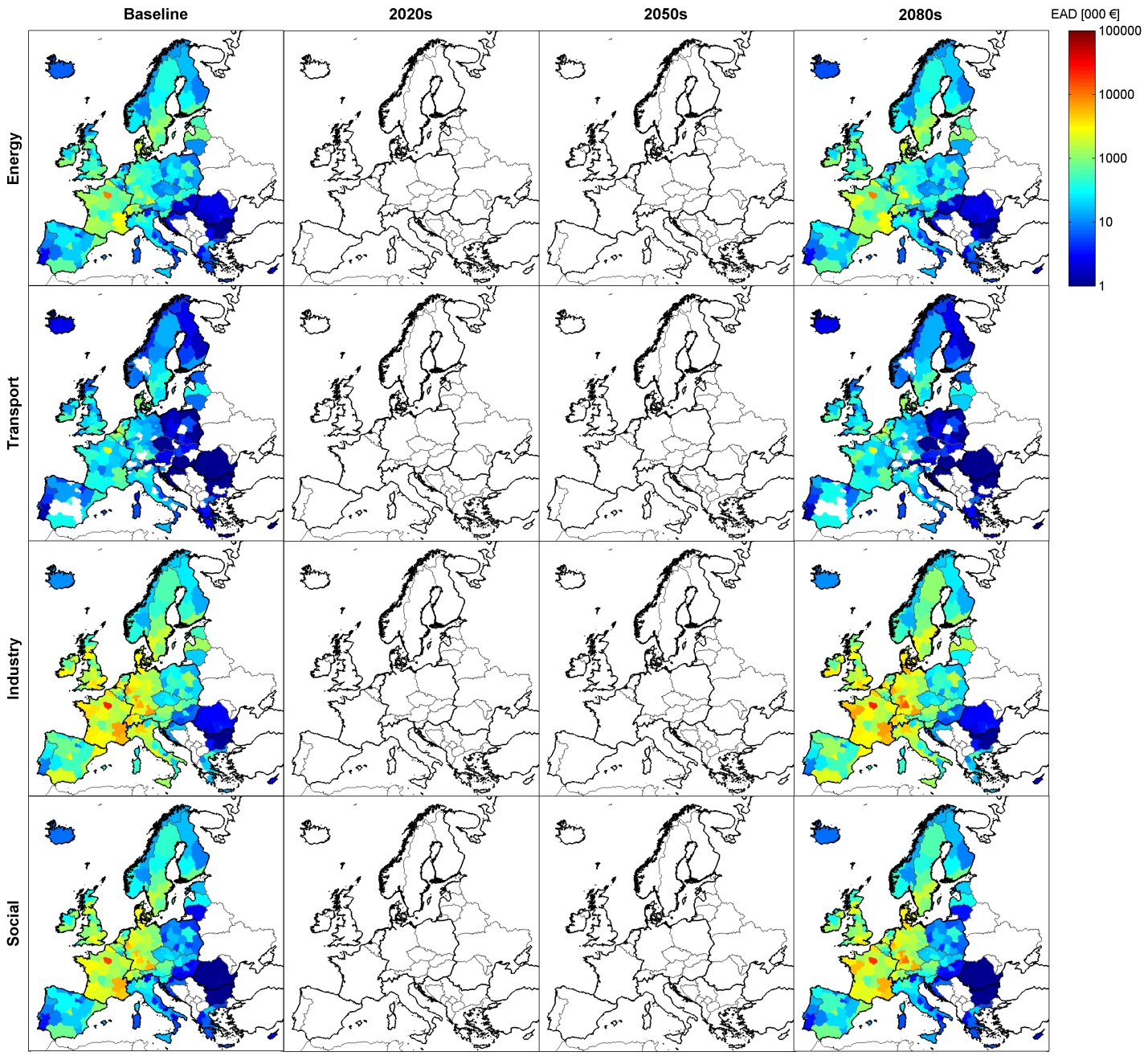
**

**Figure S11 |** Expected annual damage due to windstorms.

**
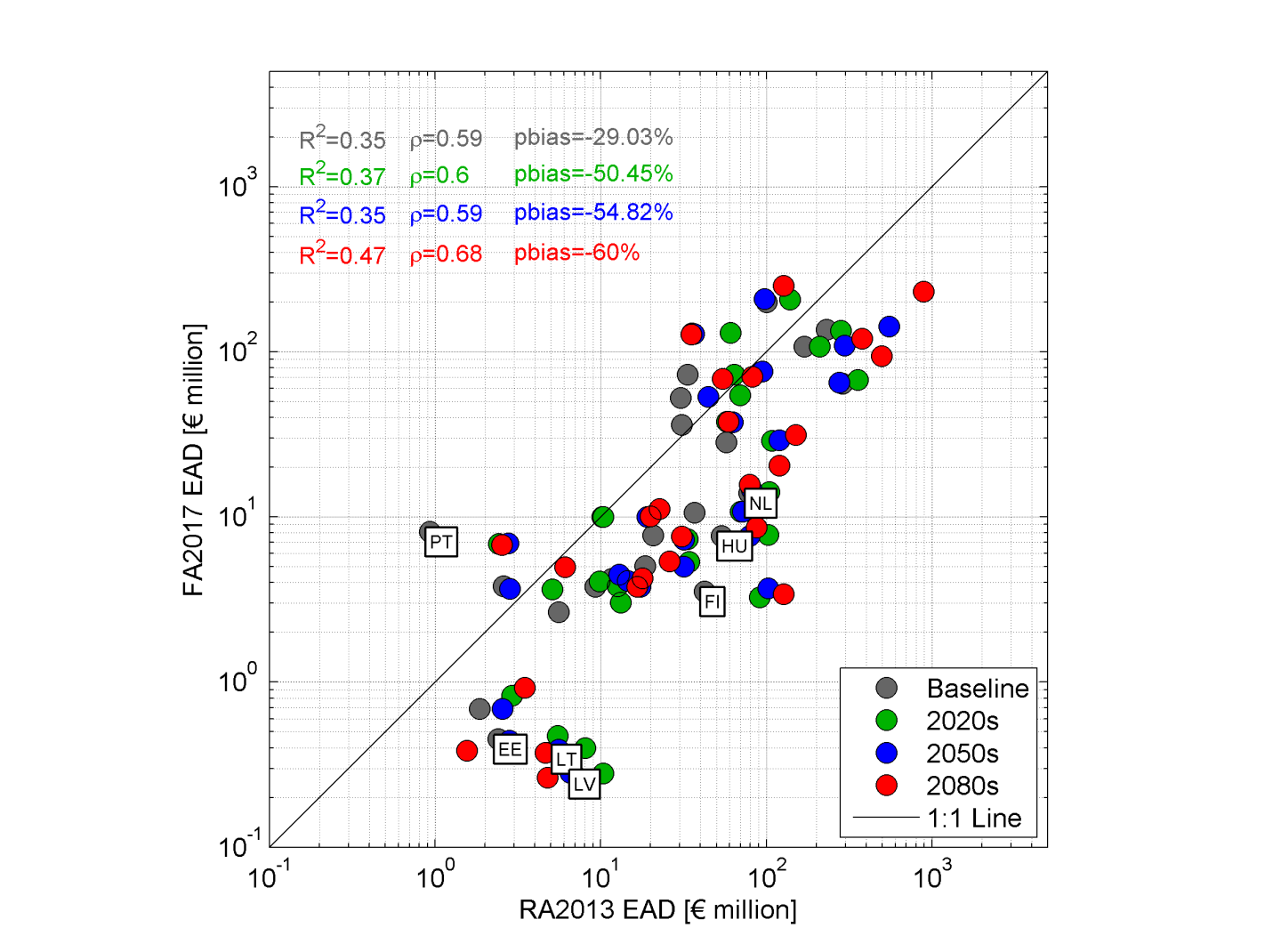
**

**Figure S12 |** Cross-comparison analysis of expected annual damage (EAD) due to river floods. Multi-sector ensemble median of EADs obtained by Rojas et al. (2013; RA2013) versus our estimates – FA2017 – (on *x*- and *y*-axis, respectively) for each country (circles) and time slice (color, as represented in legend). Labels are displayed for countries with divergences in EAD larger than 400%. Reliability coefficients synthesize the degree of agreement in terms of coefficient of determination (*R*^2^), Pearson correlation (*ρ*) and percent bias (pbias).

**References**

Batista e Silva, F., Gallego, J., Lavalle, C., 2013. A high-resolution population grid map for Europe. J. Maps 9, 16–28. doi:10.1080/17445647.2013.764830

Bebb, J, Kersey, J, 2003. Potential impacts of climate change on waste management. Environment Agency, Bristol, UK.

Bompard, E., Huang, T., Wu, Y., Cremenescu, M., 2013. Classification and trend analysis of threats origins to the security of power systems. Int. J. Electr. Power Energy Syst. 50, 50–64. doi:10.1016/j.ijepes.2013.02.008

Broekx, S., Smets, S., Liekens, I., Bulckaen, D., Nocker, L.D., 2011. Designing a long-term flood risk management plan for the Scheldt estuary using a risk-based approach. Nat. Hazards 57, 245–266. doi:10.1007/s11069-010-9610-x

Brown, S., Hanson, S., Nicholls, R.J., 2013. Implications of sea-level rise and extreme events around Europe: a review of coastal energy infrastructure. Clim. Change 122, 81–95. doi:10.1007/s10584-013-0996-9

Carmichael, C., Bickler, G., Kovats, S., Pencheon, D., Murray, V., West, C., Doyle, Y., 2012. Overheating and Hospitals - What do we know? J. Hosp. Adm. 2. doi:10.5430/jha.v2n1p1

Chandramowli, S.N., Felder, F.A., 2014. Impact of climate change on electricity systems and markets – A review of models and forecasts. Sustain. Energy Technol. Assess. 5, 62–74. doi:10.1016/j.seta.2013.11.003

Chinowsky, P.S., Price, J.C., Neumann, J.E., 2013. Assessment of climate change adaptation costs for the U.S. road network. Glob. Environ. Change 23, 764–773. doi:10.1016/j.gloenvcha.2013.03.004

Ciscar JC, Feyen L, Soria A, Lavalle C, Raes F, Perry M, Nemry F, Demirel H, Rozsai M, Dosio A, Donatelli M, Srivastava A, Fumagalli D, Niemeyer S, Shrestha S, Ciaian P, Himics M, Van Doorslaer B, Barrios S, Ibáñez N, Forzieri G, Rojas R, Bianchi A, Dowling P, Camia A, Libertà G, San Miguel J, de Rigo D, Caudullo G, Barredo J-I, Paci D, Pycroft J, Saveyn B, Van Regemorter D, Revesz T, Vandyck T, Vrontisi Z, Baranzelli C, Vandecasteele I, Batista e Silva F, Ibarreta D, 2014. Climate Impacts in Europe The JRC PESETA II Project (No. JRC87011). Luxembourg.

Corti, T., Muccione, V., Köllner-Heck, P., Bresch, D., Seneviratne, S.I., 2009. Simulating past droughts and associated building damages in France. Hydrol Earth Syst Sci 13, 1739–1747. doi:10.5194/hess-13-1739-2009

Corti, T., Wüest, M., Bresch, D., Seneviratne, S.I., 2011. Drought-induced building damages from simulations at regional scale. Nat Hazards Earth Syst Sci 11, 3335–3342. doi:10.5194/nhess-11-3335-2011

Cozzani, V., Campedel, M., Renni, E., Krausmann, E., 2010. Industrial accidents triggered by flood events: Analysis of past accidents. J. Hazard. Mater. 175, 501–509. doi:10.1016/j.jhazmat.2009.10.033

Cruz, A.M., Krausmann, E., 2013. Vulnerability of the oil and gas sector to climate change and extreme weather events. Clim. Change 121, 41–53. doi:10.1007/s10584-013-0891-4

Delpla, I., Jung, A.-V., Baures, E., Clement, M., Thomas, O., 2009. Impacts of climate change on surface water quality in relation to drinking water production. Environ. Int. 35, 1225–1233. doi:10.1016/j.envint.2009.07.001

Dobney, K., Baker, C.J., Quinn, A.D., Chapman, L., 2009. Quantifying the effects of high summer temperatures due to climate change on buckling and rail related delays in south-east United Kingdom. Meteorol. Appl. 16, 245–251. doi:10.1002/met.114

Doll, C., Klug, S., Enei, R., 2014. Large and small numbers: options for quantifying the costs of extremes on transport now and in 40 years. Nat. Hazards 72, 211–239. doi:10.1007/s11069-013-0821-9

Doll, C., Klug, S., Partzsch, I., Enei, R., Pelikan, V., Sedlacek, N., Maurer, M., Rudzikaite, L., Papanikolaou, A., Haverd, V., 2011. Adaptation strategies in the transport sector. FP7 project WEATHER Deliverable 4. Fraunhofer ISI , Karlsruhe.

EA, 2009. Investing for the future: Flood and coastal risk management in England - A long-term investment strategy. UK Environment Agency.

Ebinger, J.O., 2011. Climate Impacts on Energy Systems: Key Issues for Energy Sector Adaptation. World Bank Publications.

Ford, J.D., Pearce, T., Prno, J., Duerden, F., Ford, L.B., Beaumier, M., Smith, T., 2009. Perceptions of climate change risks in primary resource use industries: a survey of the Canadian mining sector. Reg. Environ. Change 10, 65–81. doi:10.1007/s10113-009-0094-8

Forzieri, G., Feyen, L., Russo, S., Vousdoukas, M., Alfieri, L., Outten, S., Migliavacca, M., Bianchi, A., Rojas, R., Cid, A., 2016. Multi-hazard assessment in Europe under climate change. Clim. Change 137, 105–119. doi:10.1007/s10584-016-1661-x

Hallegatte, S., Corfee-Morlot, J., 2011. Understanding climate change impacts, vulnerability and adaptation at city scale: an introduction. Clim. Change 104, 1–12. doi:10.1007/s10584-010-9981-8

Hallegatte, S., Ranger, N., Mestre, O., Dumas, P., Corfee-Morlot, J., Herweijer, C., Wood, R.M., 2010. Assessing climate change impacts, sea level rise and storm surge risk in port cities: a case study on Copenhagen. Clim. Change 104, 113–137. doi:10.1007/s10584-010-9978-3

Hjerp, P., Volkery, A., Lückge, H., Medhurst, J., Hart, K., Medarova-Bergstrom, K., Tröltzsch, J., McGuinn, J., Skinner, I., Desbarats, J., Slater, C., Bartel, A., Frelih-Larsen, A., ten Brink, P., 2012. Methodologies for Climate Proofing Investments and Measures under Cohesion and Regional Policy and the Common Agricultural Policy (A report for DG Climate).

Holub, M., Fuchs, S., 2008. Benefits of local structural protection to mitigate torrent-related hazards. WIT Press, pp. 401–411. doi:10.2495/RISK080391

Hooper, E., Chapman, L., 2012. Chapter 5 The Impacts of Climate Change on National Road and Rail Networks, in: Ryley, T., Chapman, L. (Eds.), Transport and Climate Change. Emerald Group Publishing Limited, pp. 105–136.

Ibánez-Rivas, N., 2010. Peer review of the TRANS-TOOLS reference transport model (No. JRC 60083). European Commission, Luxembourg.

Jaroszweski, D., Chapman, L., Petts, J., 2010. Assessing the potential impact of climate change on transportation: the need for an interdisciplinary approach. J. Transp. Geogr. 18.

Jonkeren, O., Rietveld, P., Ommeren, J. van, 2007. Climate Change and Inland Waterway Transport: Welfare Effects of Low Water Levels on the River Rhine. J. Transp. Econ. Policy 41, 387–411.

Jonkeren, O., Rietveld, P., Ommeren, J. van, Linde, A. te, 2013. Climate change and economic consequences for inland waterway transport in Europe. Reg. Environ. Change 14, 953–965. doi:10.1007/s10113-013-0441-7

Koetse, M.J., Rietveld, P., 2009. The impact of climate change and weather on transport: An overview of empirical findings. Transp. Res. Part Transp. Environ. 14, 205–221. doi:10.1016/j.trd.2008.12.004

Kontogianni, A., Tourkolias, C.H., Damigos, D., Skourtos, M., 2014. Assessing sea level rise costs and adaptation benefits under uncertainty in Greece. Environ. Sci. Policy 37, 61–78. doi:10.1016/j.envsci.2013.08.006

Krausmann, E., Cozzani, V., Salzano, E., Renni, E., 2011. Industrial accidents triggered by natural hazards: an emerging risk issue. Nat Hazards Earth Syst Sci 11, 921–929. doi:10.5194/nhess-11-921-2011

Langeveld, J.G., Schilperoort, R.P.S., Weijers, S.R., 2013. Climate change and urban wastewater infrastructure: There is more to explore. J. Hydrol. 476, 112–119. doi:10.1016/j.jhydrol.2012.10.021

Lehner, B., Döll, P., Alcamo, J., Henrichs, T., Kaspar, F., 2006. Estimating the Impact of Global Change on Flood and Drought Risks in Europe: A Continental, Integrated Analysis. Clim. Change 75, 273–299. doi:10.1007/s10584-006-6338-4

Leviäkangas, P, Tuominen, A, Molarius, R, Kojo, H, Schabel, J, Toivonen, S, Keränen, J, Ludvigsen, J, Vajda, A, Tuomenvirta, H, Juga, I, Nurmi, P, Rauhala, J, Rehm, F, Gerz, T, Muehlhausen, T, Schweighofer, J, Michaelides, S, Papadakis, M, Dotzek, N, Groenemeijer, P, 2011. Extreme weather impacts on transport systems (No. 168), VTT Working Papers. Finland.

Linnerud, K., Mideksa, T.K., Eskeland, G.S., 2011. The Impact of Climate Change on Nuclear Power Supply. Energy J. 32. doi:10.5547/ISSN0195-6574-EJ-Vol32-No1-6

Major, D.C., Bader, D., Leichenko, R.M., Johnson, K., Linkin, M., 2014. Projecting Future Insured Coastal Flooding Damages with Climate Change.

Middelkoop, H., Daamen, K., Gellens, D., Grabs, W., Kwadijk, J.C.J., Lang, H., Parmet, B.W. a. H., Schädler, B., Schulla, J., Wilke, K., 2001. Impact of Climate Change on Hydrological Regimes and Water Resources Management in the Rhine Basin. Clim. Change 49, 105–128. doi:10.1023/A:1010784727448

Mideksa, T.K., Kallbekken, S., 2010. The impact of climate change on the electricity market: A review. Energy Policy, Large-scale wind power in electricity markets with Regular Papers 38, 3579–3585. doi:10.1016/j.enpol.2010.02.035

Mima, S., Criqui, P., 2015. The Costs of Climate Change for the European Energy System, an Assessment with the POLES Model. Environ. Model. Assess. 20, 303–319. doi:10.1007/s10666-015-9449-3

Mitchell, J.W., 2013. Power line failures and catastrophic wildfires under extreme weather conditions. Eng. Fail. Anal., Special issue on ICEFA V- Part 1 35, 726–735. doi:10.1016/j.engfailanal.2013.07.006

Moiseyev, A., Solberg, B., Kallio, A.M.I., Lindner, M., 2011. An economic analysis of the potential contribution of forest biomass to the EU RES target and its implications for the EU forest industries. J. For. Econ., Fuelwood, timber and climate change: Insights from the forest sector modeling 17, 197–213. doi:10.1016/j.jfe.2011.02.010

Molarius, R., Könönen, V., Leviäkangas, P., Zulkarnain, Rönty, J., Hietajärvi, A.-M., Oiva, K., 2013. The extreme weather risk indicators (EWRI) for the European transport system. Nat. Hazards 72, 189–210. doi:10.1007/s11069-013-0650-x

Neumann, J.E., Price, J., Chinowsky, P., Wright, L., Ludwig, L., Streeter, R., Jones, R., Smith, J.B., Perkins, W., Jantarasami, L., Martinich, J., 2014. Climate change risks to US infrastructure: impacts on roads, bridges, coastal development, and urban drainage. Clim. Change 131, 97–109. doi:10.1007/s10584-013-1037-4

Nielsen-Pincus, M., Moseley, C., Gebert, K., 2014. Job growth and loss across sectors and time in the western US: The impact of large wildfires. For. Policy Econ. 38, 199–206. doi:10.1016/j.forpol.2013.08.010

Oven, K.J., Curtis, S.E., Reaney, S., Riva, M., Stewart, M.G., Ohlemüller, R., Dunn, C.E., Nodwell, S., Dominelli, L., Holden, R., 2012. Climate change and health and social care: Defining future hazard, vulnerability and risk for infrastructure systems supporting older people’s health care in England. Appl. Geogr., The Health Impacts of Global Climate Change: A Geographic Perspective 33, 16–24. doi:10.1016/j.apgeog.2011.05.012

Palin, E.J., Thornton, H.E., Mathison, C.T., McCarthy, R.E., Clark, R.T., Dora, J., 2013. Future projections of temperature-related climate change impacts on the railway network of Great Britain. Clim. Change 120, 71–93. doi:10.1007/s10584-013-0810-8

Paskal, C, 2010. The vulnerability of energy infrastructure to environmental change. China Eurasia Forum Q. 149–163.

Patt, A., Pfenninger, S., Lilliestam, J., 2013. Vulnerability of solar energy infrastructure and output to climate change. Clim. Change 121, 93–102. doi:10.1007/s10584-013-0887-0

Pejovic, T., Williams, V., Noland, R., Toumi, R., 2009. Factors Affecting the Frequency and Severity of Airport Weather Delays and the Implications of Climate Change for Future Delays. Transp. Res. Rec. J. Transp. Res. Board 2139, 97–106. doi:10.3141/2139-12

Petrascheck, A., 2003. The “Action plan on Flood Defence “of the International Commission for the Protection of the Rhine as an example for European Co-operation.

Pita, G.L., Pinelli, J.-P., Gurley, K.R., Hamid, S., 2013. Hurricane vulnerability modeling: Development and future trends. J. Wind Eng. Ind. Aerodyn. 114, 96–105. doi:10.1016/j.jweia.2012.12.004

Pryor, S.C., Barthelmie, R.J., 2010. Climate change impacts on wind energy: A review. Renew. Sustain. Energy Rev. 14, 430–437. doi:10.1016/j.rser.2009.07.028

Pryor, S.C., Barthelmie, R.J., Kjellström, E., 2005. Potential climate change impact on wind energy resources in northern Europe: analyses using a regional climate model. Clim. Dyn. 25, 815–835. doi:10.1007/s00382-005-0072-x

Radovic, V., Vitale, K., Tchounwou, P.B., 2012. Health Facilities Safety in Natural Disasters: Experiences and Challenges from South East Europe. Int. J. Environ. Res. Public. Health 9, 1677–1686. doi:10.3390/ijerph9051677

Rojas, R., Feyen, L., Watkiss, P., 2013. Climate change and river floods in the European Union: Socio-economic consequences and the costs and benefits of adaptation. Glob. Environ. Change 23, 1737–1751. doi:10.1016/j.gloenvcha.2013.08.006

Rübbelke, D., Vögele, S., 2011. Impacts of climate change on European critical infrastructures: The case of the power sector. Environ. Sci. Policy 14, 53–63. doi:10.1016/j.envsci.2010.10.007

Sathaye, J.A., Dale, L.L., Larsen, P.H., Fitts, G.A., Koy, K., Lewis, S.M., de Lucena, A.F.P., 2013. Rising Temps, Tides, and Wildfires: Assessing the Risk to California’s Energy Infrastructure from Projected Climate Change. IEEE Power Energy Mag. 11, 32–45. doi:10.1109/MPE.2013.2245582

Schaeffer, R., Szklo, A.S., Pereira de Lucena, A.F., Moreira Cesar Borba, B.S., Pupo Nogueira, L.P., Fleming, F.P., Troccoli, A., Harrison, M., Boulahya, M.S., 2012. Energy sector vulnerability to climate change: A review. Energy 38, 1–12. doi:10.1016/j.energy.2011.11.056

Schröter, K., Ostrowski, M., Velasco, C., Sempere Torres, D., Nachtnebel, H., Kahl, B., Beyene, M., Rubin, C., Gocht, M., 2008. Effectiveness and Efficiency of Early Warning Systems For Flash Floods (EWASE).

Schweighofer, J., 2013. The impact of extreme weather and climate change on inland waterway transport. Nat. Hazards 72, 23–40. doi:10.1007/s11069-012-0541-6

Sieber, J., 2013. Impacts of, and adaptation options to, extreme weather events and climate change concerning thermal power plants. Clim. Change 121, 55–66. doi:10.1007/s10584-013-0915-0

Stewart, M.G., Wang, X., Nguyen, M.N., 2011. Climate change impact and risks of concrete infrastructure deterioration. Eng. Struct. 33, 1326–1337. doi:10.1016/j.engstruct.2011.01.010

Suarez, P., Anderson, W., Mahal, V., Lakshmanan, T.R., 2005. Impacts of flooding and climate change on urban transportation: A systemwide performance assessment of the Boston Metro Area. Transp. Res. Part Transp. Environ. 10, 231–244. doi:10.1016/j.trd.2005.04.007

Tang, R., Clark, J.M., Bond, T., Graham, N., Hughes, D., Freeman, C., 2013. Assessment of potential climate change impacts on peatland dissolved organic carbon release and drinking water treatment from laboratory experiments. Environ. Pollut. 173, 270–277. doi:10.1016/j.envpol.2012.09.022

Tröltzsch, J., Görlach, B., Helen, L., Peter, M., Christian, S., 2012. Kosten und Nutzen von Anpassungsmaßnahmen an den Klimawandel, Analyse von 28 Anpassungsmaßnahmen in Deutschland. Umweltbundesamt, Dessau-Roßlau, Germany.

Vajda, A., Tuomenvirta, H., Juga, I., Nurmi, P., Jokinen, P., Rauhala, J., 2013. Severe weather affecting European transport systems: the identification, classification and frequencies of events. Nat. Hazards 72, 169–188. doi:10.1007/s11069-013-0895-4

van Vliet, M.T.H., Yearsley, J.R., Ludwig, F., Vögele, S., Lettenmaier, D.P., Kabat, P., 2012. Vulnerability of US and European electricity supply to climate change. Nat. Clim. Change 2, 676–681. doi:10.1038/nclimate1546

Vliet, M.T.H. van, Vögele, S., Rübbelke, D., 2013. Water constraints on European power supply under climate change: impacts on electricity prices. Environ. Res. Lett. 8, 035010. doi:10.1088/1748-9326/8/3/035010

Whitehead, P.G., Wilby, R.L., Battarbee, R.W., Kernan, M., Wade, A.J., 2009. A review of the potential impacts of climate change on surface water quality. Hydrol. Sci. J. 54, 101–123. doi:10.1623/hysj.54.1.101

Wright, L., Chinowsky, P., Strzepek, K., Jones, R., Streeter, R., Smith, J.B., Mayotte, J.-M., Powell, A., Jantarasami, L., Perkins, W., 2012. Estimated effects of climate change on flood vulnerability of U.S. bridges. Mitig. Adapt. Strateg. Glob. Change 17, 939–955. doi:10.1007/s11027-011-9354-2
